# A theoretical model of inflammation- and mechanotransduction-driven asthmatic airway remodelling

**DOI:** 10.1007/s10237-018-1037-4

**Published:** 2018-07-02

**Authors:** Michael R. Hill, Christopher J. Philp, Charlotte K. Billington, Amanda L. Tatler, Simon R. Johnson, Reuben D. O’Dea, Bindi S. Brook

**Affiliations:** 1 Centre for Mathematical Medicine and Biology, School of Mathematical Sciences, University of Nottingham, Room C25, Mathematical Sciences Building, University Park, Nottingham, NG7 2RD UK; 2 Division of Respiratory Medicine, Nottingham Biomedical Research Centre, University of Nottingham, D Floor, South Block, Queen’s Medical Centre Campus, Nottingham, NG7 2UH UK; 3 Division of Respiratory Medicine, Nottingham Biomedical Research Centre, Nottingham City Hospital, University of Nottingham, Hucknall Road, Nottingham, NG5 1PB UK; 4 Centre for Mathematical Medicine and Biology, School of Mathematical Sciences, University of Nottingham, Room C28, Mathematical Sciences Building, University Park, Nottingham, NG7 2RD UK; 5 Centre for Mathematical Medicine and Biology, School of Mathematical Sciences, University of Nottingham, Room C26, Mathematical Sciences Building, University Park, Nottingham, NG7 2RD UK

**Keywords:** Morphoelastic, Bronchoconstriction, Hyper-responsiveness, Mechanochemical, Airway smooth muscle, Multiphase

## Abstract

Inflammation, airway hyper-responsiveness and airway remodelling are well-established hallmarks of asthma, but their inter-relationships remain elusive. In order to obtain a better understanding of their inter-dependence, we develop a mechanochemical morphoelastic model of the airway wall accounting for local volume changes in airway smooth muscle (ASM) and extracellular matrix in response to transient inflammatory or contractile agonist challenges. We use constrained mixture theory, together with a multiplicative decomposition of growth from the elastic deformation, to model the airway wall as a nonlinear fibre-reinforced elastic cylinder. Local contractile agonist drives ASM cell contraction, generating mechanical stresses in the tissue that drive further release of mitogenic mediators and contractile agonists via underlying mechanotransductive signalling pathways. Our model predictions are consistent with previously described inflammation-induced remodelling within an axisymmetric airway geometry. Additionally, our simulations reveal novel mechanotransductive feedback by which hyper-responsive airways exhibit increased remodelling, for example, via stress-induced release of pro-mitogenic and pro-contractile cytokines. Simulation results also reveal emergence of a persistent contractile tone observed in asthmatics, via either a pathological mechanotransductive feedback loop, a failure to clear agonists from the tissue, or a combination of both. Furthermore, we identify various parameter combinations that may contribute to the existence of different asthma phenotypes, and we illustrate a combination of factors which may predispose severe asthmatics to fatal bronchospasms.

**Electronic supplementary material:**

The online version of this article (10.1007/s10237-018-1037-4) contains supplementary material, which is available to authorized users.

## Introduction

Asthma is a chronic lung disease characterized by inflammation, airway hyper-responsiveness (excessive bronchoconstriction in response to relatively low doses of contractile agonist; AHR) and airway remodelling. The last of these involves a series of structural changes, including thickening of the epithelial layer and subepithelial basement membrane (SBM; the collagen-dominated inner layer of the airway) and of airway smooth muscle (ASM) bundles (Holgate 2011; Brightling et al. 2012; James et al. 2012; Berair et al. 2013). While each of these three features contributes to asthma severity, how they interact is poorly understood. Most importantly, it is not clear whether AHR or remodelling are causes or consequences of the disease.

We hypothesize that while airway remodelling is initiated by inflammatory mediators, it is perpetuated by mechanical factors. The complexity of the underlying biochemical and mechanical processes, which span multiple length and timescales, makes identification of key interactions solely from biological experiments on isolated processes particularly challenging. Our aim is, therefore, to investigate the combined effect of repeated, short timescale, inflammatory episodes and associated mechanical forces, arising from ASM cell (ASMC) contraction, on long-term airway remodelling. To this end, we present a novel, quantitative mechanochemical modelling framework (informed by appropriate in vitro and in vivo studies) that integrates these processes for the first time. We use this model to elucidate emergent system dynamics and thereby identify key underlying pathogenic processes.

Although inflammation is considered to be the main process by which airway remodelling occurs, based on in vitro (Brightling et al. 2012; Dekkers et al. 2012; Noble et al. 2014 and references therein) and animal (Sjöberg et al. 2017; Alrifai et al. 2014; Silva et al. 2008; Zhu et al. 1999) studies, the causative effects of inflammation on remodelling are not fully supported by clinical trial or epidemiological data. For example, controlling inflammation with inhaled corticosteroids does not change the extent of airway remodelling or the decline of lung function with age (Guilbert et al. 2006; Strunk 2007). Moreover, airway remodelling may occur in early life in the absence of inflammation (James et al. 2012, 2009). Bronchoconstriction, in the absence of inflammation, can also promote airway remodelling (Kistemaker et al. 2014; Oenema et al. 2013; Ge et al. 2012; Grainge et al. 2011; Tatler et al. 2011). In addition to the structural changes present in asthmatic airways, there is increasing evidence of altered baseline contractile tone that is thought to be the result of the chronic presence of contractile agonist or inflammatory mediators (Brightling et al. 2002).

It is not clear how this persistent tone arises, but it could enhance AHR (Bossé et al. 2009). Additionally, intra-subject heterogeneity of tone in asthmatics has been proposed as a cause of AHR (Brown and Togias 2016). Given that the area fraction of ASM in human biopsies has been shown to increase with the degree of asthma severity (Hassan et al. 2010), altered tone and ASM mass are likely interrelated. Furthermore, airway remodelling is also associated with extracellular matrix (ECM) changes (Kuo et al. 2011) and SBM thickening (Benayoun et al. 2003).

Inflammation in the airways is the protective response to allergen challenges, and is characterized by the recruitment of inflammatory cells (such as eosinophils), activation of resident mast cells, and over-expression of cytokines and chemokines (Brightling et al. 2003). Some of the latter interact directly with ASM to trigger contraction, and/or interact with mast cells causing them to degranulate, producing histamine and other contractile agonists (Kostenis and Ulven 2006). Inflammatory cells release mediators that also have the ability to induce remodelling (e.g. TGF-$\beta$; Halwani et al. 2011). Subsequently, the inflammatory cells and cytokines are gradually cleared from the tissue. Allergen challenges in humans are typically random, but in mouse models of asthma, inflammatory and contractile agonist challenges are administered and controlled artificially.

Tissue mechanics plays a significant role in airway remodelling and bronchoconstriction. For example, mechanical strain increases ASMC proliferation (Smith et al. 1994) and contractile protein expression (Smith et al. 1997, 1995). In addition, TGF-$\beta$ is a cytokine that mediates remodelling by inducing both cell proliferation and ECM protein production (Halwani et al. 2011), as well as having a potential contractile agonist role (Desmoulière et al. 2005; Grinnell and Ho 2002; Montesano and Orci 1988). It is activated by ASMCs (Tatler et al. 2011), likely during bronchoconstriction (Oenema et al. 2013; Tatler and Jenkins 2012) via mechanical stretch from latent complexes that are tethered to the ECM and to the ASMC (Froese et al. 2016; Noble et al. 2014; Wipff et al. 2007). Moreover, bronchial epithelial cells under mechanical compression shed growth factors such as endothelin-1 (ET-1), early growth response-1 (EGR-1) and TGF-$\beta$ (Tschumperlin and Drazen 2006, 2001; Tschumperlin et al. 2004) as well as to signal lung fibroblasts to express ECM proteins in vitro (Swartz et al. 2001).

The heterogeneous micro-mechanical stress environment, generated by ASM contraction, can be quantitatively and qualitatively very different in normal versus remodelled airways (Hiorns et al. 2016). Associated mechanotransductive processes may influence ECM deposition and ASM proliferation and migration. While it is recognized that these mechanisms play a crucial role in numerous developmental, physiological and pathological processes in other diseases (Hoffman et al. 2011; Martinez-Lemus et al. 2009), we have yet to understand how these mechanisms contribute to AHR and airway remodelling in asthma.

A significant body of work focusses on models of bronchoconstriction (e.g. Hiorns et al. 2014; Eskandari et al. 2013; Politi et al. 2010; Wang et al. 2008; Latourelle et al. 2002; Lambert and Paré 1997; Macklem 1996; Lambert et al. 1993), with much less attention paid to modelling of inflammation in the airways. Of particular relevance to this work are two of our previous models: firstly, our finite-thickness continuum-based model (Hiorns et al. 2014) of agonist-initiated contraction of the airway (represented as a nonlinear fibre-reinforced elastic material), which accounts for contractile force generation at the cell-level; secondly, our spatially-averaged (ordinary differential equation; ODE) model (Chernyavsky et al. 2014) of inflammation-driven switching of ASMCs from a contractile to a proliferative phenotype. The former predicts airway calibre changes and spatial stress heterogeneities, as a result of ASM contraction and in response to pressure fluctuations that mimic tidal breathing. In the latter, resolution of inflammation is implicated as the key factor in driving ASM mass accumulation; however, this model does not account for mechanotransductive feedback from mechanical stresses arising in the constricted remodelled airway wall. Thus, we combine and extend these two models by: (i) recasting our biomechanical model of the contractile airway into a well-established morphoelastic framework, that has been widely applied to growth and remodelling of soft tissues; and (ii) coupling this to the evolution of individual tissue constituents, using a multiphase description, governed by underlying biochemical processes such as inflammation-induced ASM phenotype switching, ASM cell proliferation or recruitment and ECM deposition, all of which can depend on mechanical stresses. Importantly, this coupled model accounts for two-way feedback between inflammation-driven changes in volume fractions of individual airway constituents and their resulting mechanochemical environment.

## Methods

### Mathematical formulation

**Fig. 1:**
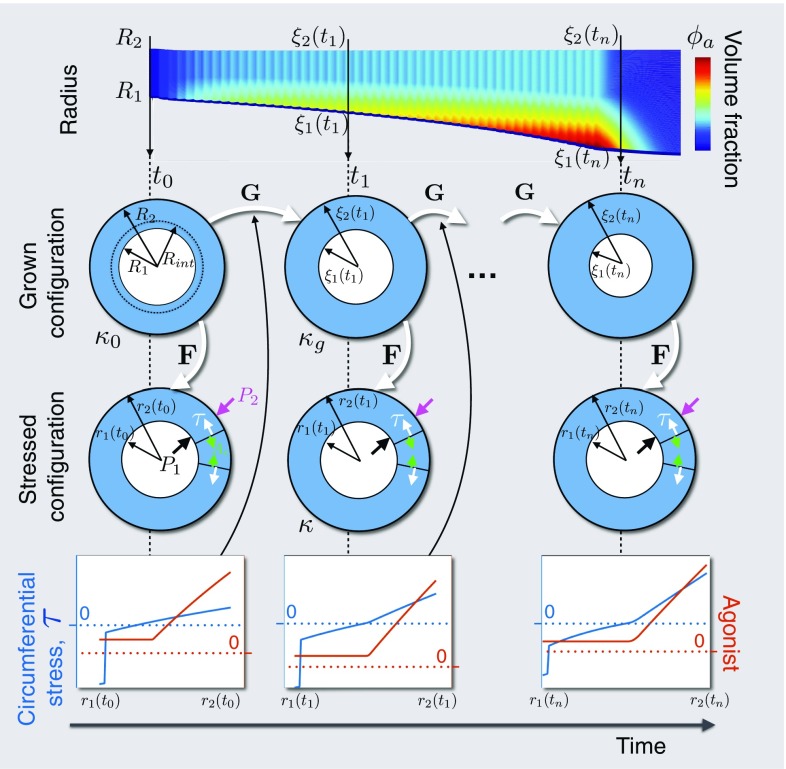
Configurations during finite growth of the airway wall. Top row: Schematic of the evolution of the airway wall geometry with time, with the colours correspoding to tissue constituent volume fraction. Middle rows: Schematics of the airway geometry in the grown and stressed configurations, at the indicated time. At initial time $t_0$, the airway wall is defined by inner radius $R_1$, interface radius $R_\mathrm{int}$, and outer radius $R_2$ in $\varvec{\kappa }_0$, the original, stress-free reference configuration. Airway wall growth is described by the mapping, $\mathbf G$, from the original configuration to $\varvec{\kappa }_g$, the grown, zero-stress configuration, defined by grown radii $\xi _1, \xi _\mathrm{int}$ (not shown), and $\xi _2$ at time $t_1$. The airway is deformed via $\mathbf F$ to configuration $\varvec{\kappa }$, the current, stressed configuration, defined by radii $r_1, r_\mathrm{int}$, and $r_2$, subject to pressure boundary conditions $P_1$ and $P_2$. Bottom row: Schematics of transmural mechanical stress distributions, generated from the active contraction and/or the elastic deformation, and contractile agonist. The mechanical stress, $\tau$, modulates certain rates in the constitutive mass balance equations, e.g. (2.18, 2.21), to influence growth, $\mathbf G$

**Fig. 2:**
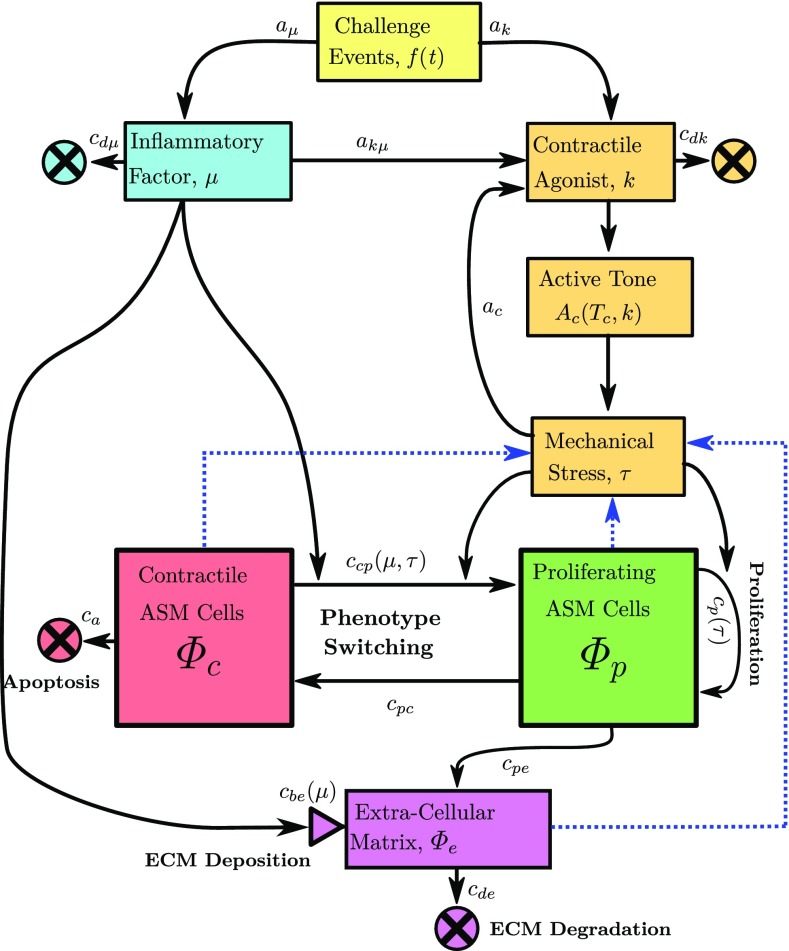
Overview of the biochemical mechanisms. Allergen or contractile agonist challenges, *f*(*t*), specified to occur at times $t_i$, drive evolution of an inflammatory factor, $\mu$, and contractile agonist concentration, *k*. The magnitude and rate of clearance of $\mu$ and *k* are determined by constants $a_\mu$, $a_k$, and $c_{\mathrm{d}\mu }, c_{\mathrm{d}k}$, respectively. Inflammation leads to *global* release of contractile agonist at rate $a_{k\mu }$. Contractile agonist induces *local* ASMC contraction, and the resulting increased mechanical stress $\tau$ releases further cytokines, with contractile agonist properties, at rate $a_\mathrm{c}$. Contractile ASMCs undergo apoptosis ($c_\mathrm{a}$) and switching to a proliferative phenotype (${c}_\mathrm{cp}$). The proliferative ASMCs divide (${c}_\mathrm{p}$) and switch to a contractile phenotype at a (high) constant rate ($c_{\mathrm{pc}}$). Both inflammation and mechanical stress drive increases (from a baseline) in the contractile to proliferative switching rate (${c}_\mathrm{cp}(\mu ,\tau )$), and increasing mechanical stress drives increases in the proliferation or recruitment rate (${c}_\mathrm{p}(\tau )$). ECM proteins degrade ($c_\mathrm{de}$) and are deposited (${c}_\mathrm{be}(\mu )$) at baseline rates during normal tissue maintenance, with the latter increasing with inflammation. Proliferating ASMCs produce additional ECM proteins (with rate $c_{\mathrm{pe}}$). Blue dotted arrows indicate how constituent volume fractions are required for computation of the mechanical stress ($\tau$), as illustrated in Fig. 1. Rate constants are given in Online Resource 2

We model the airway wall as a two-layered cylinder, representing the inner collagenous SBM and an outer (predominantly) smooth muscle layer, within which accumulation of ASM mass and ECM deposition is driven by both biochemical and mechanical stimuli (Figs. 1, 2), neglecting (for simplicity) the sub-mucosal epithelial layer. We first outline a description of the elastic deformation (Fig. 1), consisting of the balance of linear momentum together with the constitutive specification of a hyperelastic mechanical law (Sect. 2.1.1). We then define the model inputs representing biochemical stimulation (Sect. 2.1.2). Finally (Sect. 2.1.3), we construct the mass balance equations, incorporating suitable biochemical and mechanotransductive processes (Fig. 2).

#### Geometry and kinematics

The airway tissue is modelled as a mixture (Ateshian 2007; Humphrey and Rajagopal 2002; Bowen 1976; Truesdell and Toupin 1960) consisting of four phases: ASMCs in either a contractile (*c*) or a proliferative (*p*) phenotype; a collagen-dominated ECM (*e*); and an extracellular fluid (*w*) that also transports soluble nutrient (not modelled) for tissue maintenance. The outer layer is composed of multiple phases (predominantly ASMCs), and the inner layer, representing the SBM, is composed entirely of the ECM phase. Following the traditional continuum mechanics approach (Holzapfel 2000; Truesdell and Noll 1965), we assume initially (at time $t_0$) that each constituent *a* in the airway is in a (common) spatial, unstressed and unstrained reference configuration denoted $\varvec{\kappa }_{0}$, in which the position of a particle is given by $\mathbf X$. We assume that the airway is an axisymmetric thick-walled cylinder of fixed length. We define fixed polar cylindrical co-ordinates $R, \varTheta , Z$ in the radial, circumferential and axial directions, respectively, with the inner layer occupying $R_1 \leqslant R \leqslant R_\mathrm{int}$, and the outer layer $R_\mathrm{int} \leqslant R \leqslant R_2$ (Fig. 1). In Fig. 1, $\mathbf {G}$ is a topological mapping from $\varvec{\kappa }_0$ to an intermediate “grown” configuration $\varvec{\kappa }_g$, in which the position of a particle originally at $\mathbf X$ is given by $\varvec{\xi }(\mathbf X ,t)$, with cylindrical co-ordinates:

2.1 $$\begin{aligned} \xi = {\xi } \left( R, t \right) , \vartheta = \varTheta , \zeta = Z, \end{aligned}$$

where we have assumed the airway maintains axisymmetry and zero axial growth. The airway in the grown configuration $\varvec{\kappa }_g$ is deformed to a stressed configuration $\varvec{\kappa }$, and the position of a particle originally at $\varvec{\xi }$ is now at $\mathbf x (\varvec{\xi })$, with the mapping given by the deformation gradient tensor $\mathbf {F}$. The total deformation is given by using the standard multiplicative decomposition, $\mathbf {H}$ = $\mathbf {F}\mathbf {G}$. For concision, explicit dependence on time is suppressed here and throughout.

For simplicity, we impose a plane-strain approximation, axisymmetry, and zero axial displacement. Thus, the deformation from $\varvec{\kappa }_g$ to $\varvec{\kappa }$ is given by

2.2 $$\begin{aligned} r = {r}(\xi ), \theta = \vartheta , z = \zeta , \end{aligned}$$

so that the elastic deformation gradient tensor is given by

2.3 $$\begin{aligned} \mathbf {F} = \mathrm {diag} \left[ \begin{array}{ccc} \frac{\partial r}{\partial \xi },&\frac{r}{\xi },&\lambda _z \end{array} \right] . \end{aligned}$$

Assuming incompressibility, $\text {det}(\mathbf {F}) = 1$ thus gives

2.4 $$\begin{aligned} r^2 =r_\mathrm{int}^2 +\xi ^2 - \xi _\mathrm{int}^2, \end{aligned}$$

where $r_\mathrm{int} = {r}(\xi _\mathrm{int})$. The initial airway geometry (Online Resource 2) is chosen to match the bovine airways used in LaPrad et al. (2010), from which the mechanical properties are obtained. These are similar in size and structure to generation 4 human airways (Harvey et al. 2013; Coxson et al. 2008; Williamson et al. 2011). The interface radius is chosen based on human airway histology (Benayoun et al. 2003).

*Mechanical properties* We assume the tissue is a nonlinear hyperelastic heterogeneous (multiphase) anisotropic material (Bowen 1976; Truesdell and Noll 1965; Truesdell and Toupin 1960). The formulation for obtaining the constitutive mechanical relation for this type of material is given in detail by Ateshian and Ricken (2010) and Ateshian (2007). We neglect dissipative stresses and assume: that all solid constituents are constrained to move together (Humphrey and Rajagopal 2002); isothermality; electroneutrality; tissue incompressibility; that stress arises from the elastic deformation only (i.e. the growth mapping does not impart stress); and that viscous stresses are negligible.

Following Hiorns et al. (2014), we apply the commonly used additive de-coupling of the active and passive Cauchy stress tensors:

2.5 $$\begin{aligned} \mathbf {T} = -p\mathbf {1} + \mathbf {T}_\mathrm{passive} + \mathbf {T}_\mathrm{active}, \end{aligned}$$

where $\mathbf {1}$ is the identity tensor and *p* is a Lagrange multiplier enforcing incompressibility, with the passive stress given by

2.6 $$\begin{aligned} \mathbf {T}_\mathrm{passive} = 2\mathbf {F} \frac{\partial \varPsi }{\partial \mathbf {C}} {\mathbf {F}}^\mathrm{T}, \end{aligned}$$

in which we highlight that, since the mixture is constrained, $\mathbf {F}_\mathrm{a}$ = $\mathbf {F}$. In (2.6), $\varPsi$ is the strain energy function per unit volume of the mixture,

2.7 $$\begin{aligned} \varPsi = \sum \limits _{\mathrm{a}=\mathrm{p},\mathrm{c},\mathrm{e}} \varPhi _\mathrm{a} W_\mathrm{a}, \end{aligned}$$

and $W_\mathrm{a}$ is the strain energy function per unit constituent volume of constituent *a* (Ateshian 2007; Huyghe and Janssen 1997), noting that the fluid phase (*w*) does not contribute to the mechanical response of the tissue.

The contractile ASMCs and collagen-dominant ECM form continuous fibre-like structures (Ijpma et al. 2017) and therefore are modelled as two sets of helical fibres wrapped symmetrically about the airway axis (to avoid torsion). The active stress in (2.5) is given by

2.8 $$\begin{aligned} \mathbf {T}_\mathrm{active} = \varPhi _\mathrm{c} A_\mathrm{c} \mathbf {m}_{\mathrm{c}}^{(j)} \otimes \mathbf {m}_{\mathrm{c}}^{(j)}, \end{aligned}$$

where $A_\mathrm{c}$ is the contractile force density, defined as the force generated by the contractile ASMCs per unit area of constituent (Brook et al. 2010) and $\mathbf {m}_{\mathrm{c}}^{(j)}$ is the contractile ASMC fibre orientation vector (see Online Resource 1.1 for further details).

*Specification of the constitutive mechanical response for the airway wall constituents* In our previous work (Hiorns et al. 2014), the airway wall is modelled as a composite material of ASMCs and ECM, in which two families of fibres are embedded in an isotropic ground matrix, thus giving an anisotropic response. Fibre recruitment is modelled phenomenologically with an exponential dependence on stretch. Here, we de-couple the mechanical responses of the proliferating ASMCs, modelled as an isotropic neo-Hookean material; the contractile ASMCs, modelled as the fibre-embedded material described above, and the ECM, modelled similarly under the additional assumption that collagen fibres bear load only after being extended beyond the recruitment stretch, $\lambda _{u}$ (Hiorns et al. 2014; Hill et al. 2012; Robertson et al. 2011; Holzapfel and Ogden 2010). The strain energy functions for each of the constituents are given in Online Resource 1.2.

We introduce a scalar quantity $\tau$ representing the mechanical stress along the contractile ASMC fibre directions, given by

2.9 $$\begin{aligned} \tau = \frac{1}{2}\sum _{j=1,2} \mathbf {T}:\mathbf {m}_{\mathrm{c}}^{(j)}\otimes \mathbf {m}_{\mathrm{c}}^{(j)}, \end{aligned}$$

which is used in the mass balance equations in Sect. 2.1.3 to elicit the mechanotransductive responses. The degree of contraction is directly related to the amount of agonist bound to the relevant contractile ASMC receptors. We therefore assume that the contractile force density, $A_\mathrm{c}$ in (2.8), is a function of contractile agonist concentration, *k*, saturating as follows:

2.10 $$\begin{aligned} A_\mathrm{c} = T_\mathrm{c} \frac{k^n}{K_d+k^n}, \end{aligned}$$

where $T_\mathrm{c}$ is a measure of hyper-responsiveness, $K_d$ represents the ratio of the dissociation rate of the ligand-receptor complex to its association rate, and *n* is the Hill coefficient, describing cooperativity.

*Balance of linear momentum* In addition to the assumptions stated above, we further assume that body forces are negligible and inertial terms may be neglected due to slow timescales associated with quasi-static deformation. Therefore, in mechanical equilibrium, conservation of linear momentum requires

2.11 $$\begin{aligned} \nabla \cdot \mathbf {T} = \mathbf {0}. \end{aligned}$$

Under the assumption of no torsion and plane strain, (2.11) reduces to

2.12 $$\begin{aligned} \frac{\partial T_{rr}}{\partial r} + \frac{T_{rr}-T_{\theta \theta }}{r} = 0, \end{aligned}$$

where $T_{rr}$ is the radial component and $T_{\theta \theta }$ the circumferential component of the Cauchy stress.

*Boundary conditions for the elastic deformation* For the elastic deformation, pressure boundary conditions are specified at the inner and outer radii, and continuity of radial displacements and stress at the interface, so that

2.13a $$\begin{aligned} {T}_{rr}({r_1})= & {} - P_1, \end{aligned}$$

2.13b $$\begin{aligned} {r}^{(i)}(\xi _\mathrm{int})= & {} {r}^{(o)}(\xi _\mathrm{int}) \equiv r_\mathrm{int}. \end{aligned}$$

2.13c $$\begin{aligned} {T}_{rr}^{(i)}(r_\mathrm{int})= & {} {T}_{rr}^{(o)}(r_\mathrm{int}), \end{aligned}$$

2.13d $$\begin{aligned} {T}_{rr}({r_2})= & {} - P_2. \end{aligned}$$

#### Model inputs

We assume that a series of transitory allergen challenges drives a global inflammatory response that represent an influx of inflammatory cells, such as eosinophils, into the airway tissue, resulting in a cumulative inflammatory status denoted $\mu$. The challenges may be administered (e.g. in chronic asthma mouse models), or occur naturally. Therefore, $\mu$ evolves according to

2.14 $$\begin{aligned} \frac{\mathrm{d}\mu }{\mathrm{d}t} = a_\mu f(t) - c_{\mathrm{d}\mu } \mu , \end{aligned}$$

where $c_{\mathrm{d}\mu }$ is the inflammation decay rate representing clearance of inflammatory cytokines or inflammatory cell apoptosis, and $a_\mu$ the magnitude, and *f*(*t*) denotes the timing of events given by (2.16).

Inflammation drives a local activation of mast cells and bronchoconstrictive mediators (represented by the concentration *k*) such as histamine or acetylcholine (Pelaia et al. 2008). The agonist induces active contraction of the ASM, which leads to airway narrowing together with associated local airway wall stresses. We assume that the local tensile stress, $\tau$ (2.9), induces activation of latent TGF-$\beta$; this cytokine also acts as a contractile agonist and therefore contributes to *k*. We also consider cases where local compressive stress drives remodelling. These cases represent compression-induced epithelial-cell-mediated expression of EGR-1 or ET-1, noting that, here, we do not model the epithelial cells directly.

As with inflammation, the frequency of contractile agonist challenges may be specified as model input to represent, for instance, artificial methacholine challenges in animals (Lauzon and Bates 2000; Gunst et al. 1988) or humans (Grainge et al. 2011). Contractile agonist, *k*, thus evolves over time according to

2.15 $$\begin{aligned} \frac{\mathrm{d}k}{\mathrm{d}t} = a_k f(t) - c_{\mathrm{d}k}k + a_{k\mu }\mu + a_\mathrm{c}\tau H(\tau ), \end{aligned}$$

where $c_{\mathrm{d}k}$ is the agonist clearance rate, $a_k$ the magnitude of administered agonist stimuli, $a_{k\mu }$ the rate of inflammation-induced agonist activation, and $a_\mathrm{c}$ the rate at which the stress $\tau$ induces agonist release. The Heaviside step function *H* ensures that only tensile stresses release *k* (Wipff et al. 2007).

By setting $a_\mu =0$ or $a_k=0$ in 2.14 and 2.15, *f*(*t*) represents either contractile agonist- or inflammatory-only challenges, respectively. The challenges are represented by a series of Gaussian peaks

2.16 $$\begin{aligned} f(t) = \frac{1}{\sqrt{2\pi \sigma ^2}}\sum \limits _{i=1}^N \left[ \text {exp}^{-d \left( t-t_i \right) ^2 / 2\sigma ^2} \right] , \end{aligned}$$

where $t_i$ is a vector of *N* event times, and *d* and $\sigma$ are constants (Chernyavsky et al. 2014).

**Fig. 3:**
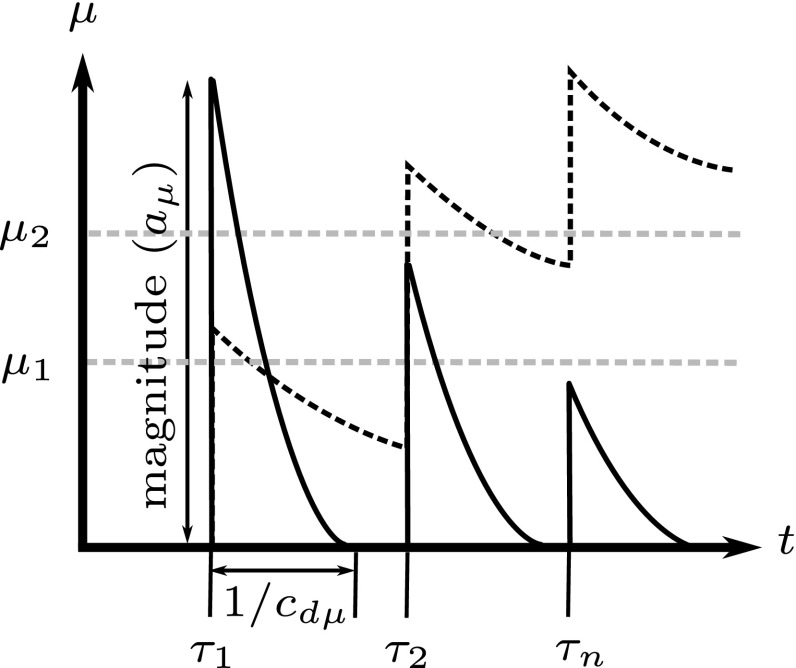
Inflammation levels. Inflammatory status dynamics induced by a series of environmental stimuli, illustrating the parameters $\mu _1, \mu _2, a_\mu , c_{\mathrm{d}\mu }$, and $t_i$, noting that for periodic events, $t_{i+1}-t_i$ = $\varDelta t$ and $\omega$ = 1/$\varDelta t$. The solid line represents relatively fast inflammatory clearance (high $c_{\mathrm{d}\mu }$), while the dotted line represents slow clearance

#### Tissue growth

Under the assumption of axisymmetry, in cylindrical polar co-ordinates, the local density, $\rho _\mathrm{a}$, of each constituent ($a = c, p, e, w$) evolves according to:

2.17 $$\begin{aligned} \frac{\partial \rho _{\mathrm{a}}}{\partial t} + v\frac{\partial \rho _\mathrm{a}}{\partial \xi }+\rho _\mathrm{a}\frac{1}{\xi }\frac{\partial (\xi v)}{\partial \xi } = s_{\mathrm{a}} \end{aligned}$$

where $\rho _\mathrm{a}$ and the (constrained mixture) radial growth velocity, *v*, are functions of the grown radius, $\xi$ (Fig. 1), and time, *t*; $s_{\mathrm{a}}$ = $s_{\mathrm{a}}\left( \rho _\mathrm{c}, \rho _{\mathrm{p}}, \rho _\mathrm{e} \right)$ represents the constituent-dependent source/sink terms, specified in detail below.

We assume that the density of contractile ASMCs, $\rho _\mathrm{c}$, evolves through switching to and from a proliferative phenotype, $\rho _\mathrm{p}$ (Fig. 2; Naveed et al. 2017; Wright et al. 2013; Hirota et al. 2009). The definition of ASMC phenotype is based on the observable function of the cells arising from expression of intracellular proteins, e.g. proliferative ASMCs exhibiting decreased expression of contractile proteins (Wright et al. 2013). As in our previous model (Chernyavsky et al. 2014), the rate of switching is governed by the inflammatory status, $\mu$, but here we additionally assume that switching can also be driven by the local fibre mechanical stress $\tau$. Thus,

2.18 $$\begin{aligned} s_\mathrm{c} = c_{\mathrm{pc}} \rho _\mathrm{p} - c_\mathrm{a} \rho _\mathrm{c}^2 - c_\mathrm{cp}\left( \mu , \tau \right) \rho _\mathrm{c} , \end{aligned}$$

where $c_{\mathrm{pc}}$ and $c_\mathrm{a}$ are positive constants, the first two terms representing switch back from the proliferative phenotype and apoptosis, respectively, the combination of which provides a logistic growth representation. $c_\mathrm{cp}$ represents the inflammation- and stress-modulated rate of switching to the proliferative phenotype given by

2.19 $$\begin{aligned} \begin{aligned} c_\mathrm{cp}(\mu ,\tau ) =&\, c_{\mathrm{c}0} + \left( c_{\mathrm{c}1} - c_{\mathrm{c}0} \right) H(\mu -\mu _1) \\&+ \left( c_{\mathrm{c}2} - c_{\mathrm{c}1} \right) H(\mu -\mu _2) + c_{\mathrm{c}\tau }(\tau ) \tau , \end{aligned} \end{aligned}$$

where $c_{\mathrm{c}0}, c_{\mathrm{c}1}, c_{\mathrm{c}2}$ and $c_{\mathrm{c}\tau }$ are positive constants. The Heaviside functions are used to divide the inflammation into three levels: healthy, mild, and severe, which are characterized by the thresholds $\mu _1$ and $\mu _2$ (Fig. 3). The final term represents local mechanical stress-induced switching:

2.20 $$\begin{aligned} c_{\mathrm{c}\tau }(\tau )= {\left\{ \begin{array}{ll} c_\mathrm{cp}^f H(\tau ) &{} \text {if tension induces switching}, \\ c_\mathrm{cp}^f H(-\tau ) &{} \text {if compression induces switching}. \end{array}\right. }\nonumber \\ \end{aligned}$$

Increases in proliferative ASMC density, $\rho _\mathrm{p}$, arise via proliferation and phenotype switching. Thus, proliferative ASMC turnover, $s_\mathrm{p}$, is represented by

2.21 $$\begin{aligned} s_\mathrm{p} = \left( c_\mathrm{p}(\tau ) - c_{\mathrm{pc}} \right) \rho _\mathrm{p} + c_\mathrm{cp}({\mu },\tau ) \rho _\mathrm{c}, \end{aligned}$$

where the proliferation rate ($c_\mathrm{p}(\tau )$) is modulated directly by the local stress $\tau$ according to

2.22 $$\begin{aligned} {c}_\mathrm{p}(\tau ) = c_{\mathrm{p}0} + c_{\mathrm{p}\tau } \tau , \end{aligned}$$

representing baseline and a (tensile or compressive) stress-driven proliferation, of identical form to (2.20) but with rate constant $c_\mathrm{p}^f$.

ECM turnover is modelled via

2.23 $$\begin{aligned} s_\mathrm{e} = c_\mathrm{pe} \rho _\mathrm{p} + {c}_\mathrm{be}(\mu ) - c_\mathrm{de} \rho _\mathrm{e}, \end{aligned}$$

where the first term represents ECM synthesis by the proliferative ASMCs, e.g. via ASMC-mediated release of active TGF-$\beta$ that induces ASMCs to synthesize collagen (Coutts et al. 2001); the second term represents modification of ECM by inflammatory mediators, e.g. via mast cell activation mediated by MMP-1; and the third term, linear degradation. The inflammation-driven ECM deposition is given by

2.24 $$\begin{aligned} {c}_\mathrm{be}({\mu })= & {} c_{\mathrm{e}0} + \left( c_{\mathrm{e}1} - c_{\mathrm{e}0} \right) H(\mu -\mu _1) \nonumber \\&+ \left( c_{\mathrm{e}2} - c_{\mathrm{e}1} \right) H(\mu -\mu _2), \end{aligned}$$

where, again, the Heaviside function is used to separate the three inflammation levels.

For simplicity, each of the constituents is considered intrinsically incompressible, i.e. their true densities ($\rho _\mathrm{a}^\mathrm{T}$) remain constant in space and time (Ateshian 2011), and we assume that the true densities of the ASMCs and ECM are equal, a reasonable assumption in general (Gleason et al. 2004). In the following, we work in terms of the volume fraction, defined by $\varPhi _\mathrm{a}=\rho _\mathrm{a}/\rho ^\mathrm{T}_\mathrm{a}$, and correspondingly write the source/sink terms as $S_\mathrm{a}=s_\mathrm{a}/\rho ^\mathrm{T}_\mathrm{a}$. Assuming no voids, we obtain

2.25 $$\begin{aligned} \sum _\mathrm{a} \varPhi _\mathrm{a} = 1. \end{aligned}$$

Equation (2.17), together with the definition of volume fraction and the source/sink terms, is re-expressed as

2.26 $$\begin{aligned} \frac{\partial \varPhi _\mathrm{a}}{\partial t} + v \frac{\partial \varPhi _\mathrm{a}}{\partial \xi } + \varPhi _\mathrm{a} \left( \frac{1}{\xi } \frac{ \partial }{\partial \xi }\left( \xi v\right) \right) = S_{\mathrm{a}} \end{aligned}$$

where the volume fractions, $\varPhi _\mathrm{a}$, are functions of the grown radius, $\xi$ (Fig. 1), and time, *t*, and $a = c,p,e$. As in Gleason et al. (2004), we assume that a constant and uniform tissue hydration is maintained, such that $\varPhi _w$ = 0.70. Summation of (2.26), together with (2.25), gives the mass balance equation for the entire mixture as

2.27 $$\begin{aligned} \frac{1}{\xi } \frac{ \partial }{\partial \xi }\left( \xi v\right) = \left( {S_{\mathrm{c}} + S_\mathrm{p} + S_{\mathrm{e}}}\right) /\left( {1 - \varPhi _w}\right) = q. \end{aligned}$$

We note that: $\varPhi _\mathrm{e}$ is obtained using (2.25); $S_{\mathrm{c}}, S_\mathrm{p}$ and $S_{\mathrm{e}}$ are functions of $\varPhi _\mathrm{c}, \varPhi _\mathrm{p}$ and $\varPhi _\mathrm{e}$. Thus (2.25), (2.27) and (2.26), together with initial conditions on $\varPhi _\mathrm{a}$ (see Online Resource 2), $\xi$ and boundary conditions on *v*, completely specify the time-evolving growth mapping $\mathbf G$, provided that the mechanical stress state can be computed (Fig. 1; Sect. 2.1.1).

*Initial and boundary conditions on tissue growth* During normal tissue maintenance, in the absence of inflammation or administered contractile agonist, we assume that ASM proliferation, recruitment and apoptosis, and ECM degradation and deposition in the airway wall balance to generate a homeostatic state. The non-trivial homogeneous steady state for (2.26), for which $v=0$, is given by

2.28a $$\begin{aligned} \varPhi _\mathrm{p}^*= & {} \frac{c_\mathrm{p}c_{\mathrm{c}0}^2}{c_\mathrm{a}(c_\mathrm{pc}-c_{\mathrm{p}0})^2\rho _\mathrm{p}^\mathrm{T}}, \end{aligned}$$

2.28b $$\begin{aligned} \varPhi _\mathrm{c}^*= & {} \frac{c_{\mathrm{p}0} c_{\mathrm{c}0}}{c_\mathrm{a}(c_{\mathrm{p}c}-c_{\mathrm{p}0})\rho _\mathrm{c}^\mathrm{T}}, \end{aligned}$$

2.28c $$\begin{aligned} \varPhi _\mathrm{e}^*= & {} \frac{c_{\mathrm{e}0} + c_\mathrm{pe}\varPhi _\mathrm{p}^*\rho _\mathrm{p}^\mathrm{T}}{c_\mathrm{de}\rho _\mathrm{e}^\mathrm{T}}, \end{aligned}$$

where we have made use of (2.18), (2.21), and (2.23). A linear stability analysis of (2.26) ensures *a priori* that the steady state (2.28) is stable. Hence, we impose this homeostatic steady state as the initial condition

2.29 $$\begin{aligned} {\varPhi }_\mathrm{a}(\xi , 0) = \varPhi _\mathrm{a}^*, \qquad a = c,p,e. \end{aligned}$$

A zero flux condition is imposed at the inner wall and at the interface between the two layers, which in cylindrical polar co-ordinates is given by

2.30a $$\begin{aligned} {v}(\xi _1) \frac{\partial \varPhi _\mathrm{a}}{\partial \xi } \biggm |_{\xi _1}= & {} 0, \end{aligned}$$

2.30b $$\begin{aligned} {v}(\xi _\mathrm{int}) \frac{\partial \varPhi _\mathrm{a}}{\partial \xi } \biggm |_{\xi _\mathrm{int}}= & {} 0. \end{aligned}$$

In order to solve the ODE (2.27) representing growth in each layer, a boundary condition must be specified on the velocity. However, because (2.27) is first order, one is unable to specify the velocity at both the inner and outer boundaries of each layer. We therefore set the radial velocity to zero on the outer wall, so that all growth occurs inwardly (this choice is discussed further in Sect. 4). Additionally, we require the velocity and displacement at the interface of the two layers to be continuous. Hence, we have

2.31a $$\begin{aligned} {\xi }(R_2)= & {} \xi _2 = R_2, \end{aligned}$$

2.31b $$\begin{aligned} {\xi }(R_\mathrm{int}^{(i)})= & {} {\xi }(R_\mathrm{int}^{(o)}) = \xi _\mathrm{int}, \end{aligned}$$

2.31c $$\begin{aligned} {v}(\xi _2)= & {} 0, \end{aligned}$$

2.31d $$\begin{aligned} {v}(\xi _\mathrm{int}^{(i)})= & {} {v}(\xi _\mathrm{int}^{(o)}), \end{aligned}$$

where the superscripts (*i*) and (*o*) denote limiting values taken from the inner and outer layers, respectively.

*Inflammatory or agonist challenge protocol* Episodes of inflammation-inducing allergen or contractile agonist challenges are represented by (2.16) in (2.14) or (2.15), respectively. Simulations are performed over a 1000 day interval, with the challenges confined to the first 50 days, thereby allowing a long resolution period to investigate the effects of the challenges on long-term airway remodelling post-challenge. The numerical solution method is outlined in Online Resource 1.3.

### Determination of material parameters

The passive material parameters are determined by fitting (2.12) to the quasi-static pressure-radius inflation data in LaPrad et al. (2010) via nonlinear regression using $\mathtt {lsqcurvefit.m}$ in MATLAB, with termination tolerances set to default values. Material parameters are given in Online Resource 2, and the fit to the data (R$^2$=0.9978) is depicted in the results below. The active response is determined by selecting values for $T_\mathrm{c}$ that qualitatively matched the active pressure-radius curves given in the work of Hiorns et al. (2014) for similar values of contractile agonist, *k*. All model parameter values and their descriptions are provided in Online Resource 2.

**Fig. 4:**
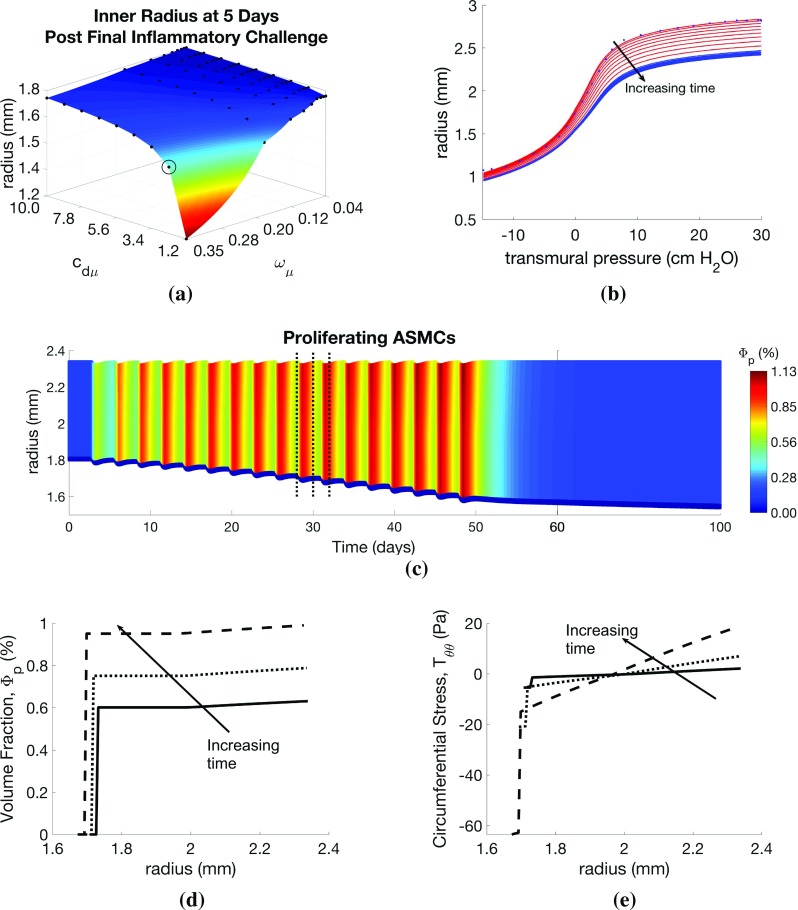
Effect of inflammatory challenges on airway remodelling and mechanics. Variation in **a** remodelled geometry with inflammation frequency ($\omega _\mu$) and resolution ($c_{\mathrm{d}\mu }$) parameter values. Illustrative results are evaluated at the circled point on the surface: **b** pressure–radius curve (red lines correspond to points during challenge; blue lines indicate resolution period; blue dots indicate data of LaPrad et al. 2010); **c** volume fraction of proliferative ASMCs ($\varPhi _\mathrm{p}$) as a function of radius and time, **d** $\varPhi _\mathrm{p}$ and **e** $T_{\theta \theta }$ as functions of radius at days 28, 30, and 32 (indicated by dotted lines in **c**). Additional plots are provided in Online Resources 4 and 5

**Fig. 5:**
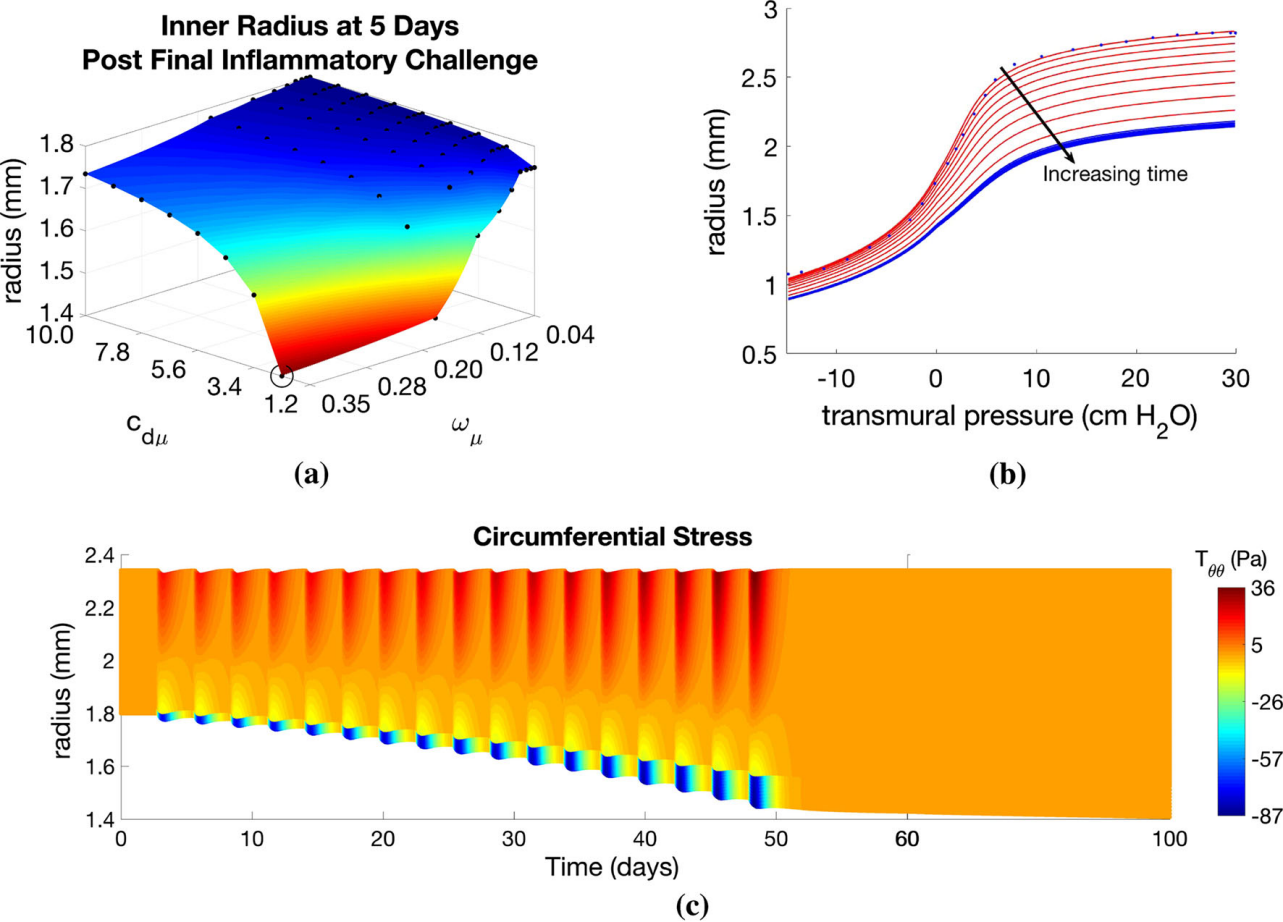
Effect of inflammatory challenge-induced subepithelial basement membrane thickening on airway remodelling and mechanics. Variation in **a** remodelled geometry with inflammation frequency ($\omega _\mu$) and resolution ($c_{\mathrm{d}\mu }$) parameter values. Illustrative results are evaluated at the circled point on the surface: **b** pressure–radius curve (red lines correspond to points during challenge; blue lines indicate resolution period; blue dots indicate data of LaPrad et al. 2010) and **c** circumferential stress ($T_{\theta \theta }$) as a function of radius and time. Results are similar to Fig. 4, except here, inflammation also drives SBM thickening

## Results

To investigate the behaviour of our novel mechanochemical morphoelastic model, we first performed a one-at-a-time sensitivity study (Online Resource 3) on selected parameters of most relevance to airway remodelling. The analysis showed that remodelling was most sensitive to the magnitude and resolution rate of inflammation, during inflammatory challenges. However, due to the possible effect of combination of the large number of parameters in this model (see Online Resource 2) and the large differences in remodelling that occur between inflammation and contractile agonist challenges, we chose to perform a series of paired parameter explorations. These are used to investigate the effect of repeated inflammatory episodes, and mechanical forces that arise from repeated ASM contractions, on long-term airway remodelling and effective mechanical properties of the airway. Parameters are set to default values unless varied in the simulations. Results are discarded for parameter choices that lead to airway growth or contraction completely into the lumen, i.e. the inner radius decreases to zero. Unless otherwise specified, remodelling occurs in the outer layer only.

*Effect of inflammatory challenges* In our first set of simulations, we apply only inflammatory challenges ($a_k =0$), mimicking regular allergen exposures in experimental studies, and explore the response to changes in challenge frequency, $\omega _{\mu }$, and inflammation resolution rate, $c_{\mathrm{d}\mu }$. From an initial inner radius of 1.8mm, we observe inward airway remodelling towards the lumen, as depicted in Fig. 4a, showing the inner radius at 5 days post-final challenge for each parameter pair. In particular, we observe a “switch” effect, in which the response is insensitive to increases in $\omega _\mu$ and decreases in $c_{\mathrm{d}\mu }$ for a large region of parameter space, but dramatic increases in remodelling occur beyond a threshold parameter set. Contractile agonist retention time, defined as the number of days between the final inflammatory or agonist challenge and the reduction in the total amount of agonist in the airway cross-section to near zero (< 1x10$^{-6}$), remains rather low for relatively high $\omega _\mu$ and low $c_{\mathrm{d}\mu }$, and is relatively insensitive to $\omega _{\mu }$, but we observe a threshold in $c_{\mathrm{d}\mu }$ below which the agonist resolution time increases abruptly to 15 days from a baseline of approximately 10 days.

Detailed results for a specific parameter choice (highlighted by circles in Fig. 4a) are shown in Fig. 4b–e. Static pressure-radius curves are computed for time points during and following inflammatory challenges (Fig. 4b). For these parameter choices, the contractile agonist concentration remains low, since the rate of inflammation-induced agonist release is relatively low, and very little ASMC contraction occurs. During challenges, the pressure-radius curve shifts downward as the airway thickens due to inflammation-induced remodelling. Following challenges, the curves continue to shift slightly downwards, due to a small amount of post-challenge remodelling, prior to a return to the steady state.

The total amount of ASM and ECM increases during inflammatory challenges as ASMCs switch from contractile to proliferative phenotype, and their volume fractions relative to that of ECM increase over time (Fig. 4c, d). Relatively low mechanical stresses arise during challenges for this parameter set, as inflammation induces only a small amount of contractile agonist release, and hence contraction is also minimal. We observe compressive radial stresses in the airway mid-wall. In the circumferential direction, the stresses are found to be tensile towards the outer edge of the airway wall and compressive near the lumen (Fig. 4e). Mechanical stresses in the axial direction are compressive due to tissue incompressibility (Online Resource 5).

*Effect of inflammatory challenge-induced ECM changes in the subepithelial basement membrane* Next, to model SBM thickening associated with asthma, we allow for inflammation-induced ECM deposition and degradation in the SBM, as well as in the outer layer, by setting $c_{\mathrm{e}0}, c_{\mathrm{e}1}, c_{\mathrm{e}2}$ and $c_\mathrm{de}$ in the inner layer to the default values given (for the outer layer). The addition of inflammation-induced ECM deposition results in increased inward remodelling (Fig. 5a), which, in turn, decreases effective airway compliance, as shown by modified pressure–radius curves (Figs. 5b, c, f, 4b). Compressive circumferential stresses in the SBM are correspondingly reduced (Fig. 5c), due to increased cross-sectional area. In contrast, peak tensile circumferential stresses in the outer layers are greater with SBM thickening, presumably as a result of a thicker, stiffer inner layer and therefore an effectively stiffer airway.

**Fig. 6:**
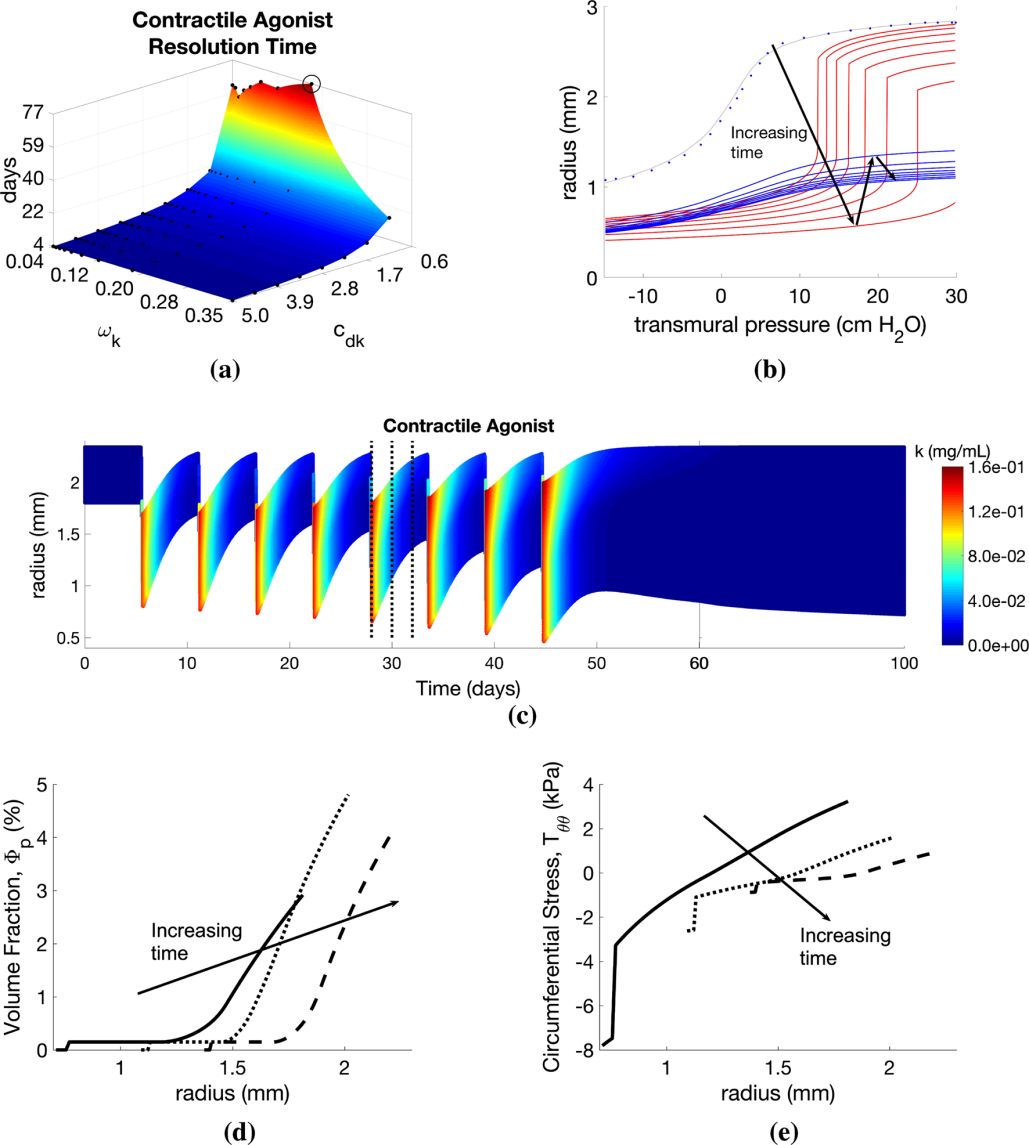
Effect of contractile agonist challenges on airway remodelling and mechanics. Variation in **a** agonist resolution rate with contractile agonist frequency ($\omega _k$) and resolution ($c_{\mathrm{d}k}$) parameter values. Illustrative results evaluated at the circled point on the surface: **b** pressure–radius curve (red lines correspond to points during challenge; blue lines indicate resolution period; blue dots indicate data of LaPrad et al. 2010), **c** contractile agonist concentration (*k*) as a function of radius and time, **d** $\varPhi _\mathrm{p}$ and (e) $T_{\theta \theta }$ as functions of radius at days 28, 30, and 32 (indicated by dotted lines on **c**). Additional plots are provided in Online Resources 4 and 5

*Effect of contractile agonist challenges* Here, we apply only contractile agonist challenges ($a_{\mu }=0$), mimicking regular methacholine exposures in experimental animal studies. We highlight that, in these simulations, all remodelling is driven by mechanotransduction, i.e. by local, stress-mediated phenotype switching. We find that increasing the agonist challenge frequency ($\omega _k$) and decreasing the contractile agonist resolution rate ($c_{\mathrm{d}k}$) lead to increased remodelling at 5 days post-challenge. As in the inflammatory-only challenges, severe remodelling is observed only beyond a threshold parameter set. Contractile agonist resolution time increases with decreasing agonist resolution rate, $c_{\mathrm{d}k}$, with a threshold below which there is a dramatic change in resolution time from a baseline of approximately 5 days to over 60 days (Fig. 6a). The dramatic increase in resolution time is a result of the build-up of agonist concentration demonstrated by nonzero agonist concentrations at the end of each challenge (Online Resource 5). Similar to the inflammation-only challenges, the contractile agonist resolution time is relatively insensitive to the challenge frequency, $\omega _k$.

Despite the reduced ECM volume fraction in the outer part of the airway (see Online Resource 5), the overall effective stiffness of the remodelled airway is increased during contractile agonist challenges, as indicated by the continued downshift in pressure–radius curves at high transmural pressures (Fig. 6b). To inflate the airway, a relatively high transmural pressure is required to overcome the increased contractile forces in the strongly contracted state, with a strong downward shift at all pressures, and the appearance of a significantly more compliant portion that shifts towards the right. When the pressure is great enough to cause stretches that exceed the recruitment threshold (see Sect. 2.1.1 and Online Resource 1.2), there is a very rapid increase in the recruitment of ECM. Tissue growth (increased airway thickness) also contributes to the observed downward shift. The separation of the effects of contractile agonist and tissue growth becomes clear following challenges, where the compliant portion of the curve disappears (first curve following challenges, Fig. 6b), and the high pressure portion of the pressure-radius curves shifts downwards as remodelling continues post-challenge.

As contractile agonist challenges cause the airways to contract (Fig. 6c), local ASMC phenotype switching (Fig. 6d) and local release of contractile agonist increase with increasing tensile circumferential stress in the outer part of the airway wall (Fig. 6e) that arises from the agonist-induced contraction. Small amounts of locally activated residual contractile agonist continue to drive remodelling post-challenge, generating a feedback loop that slows agonist clearance (Fig. 6a), accounting for build-up in agonist concentration.

**Fig. 7:**
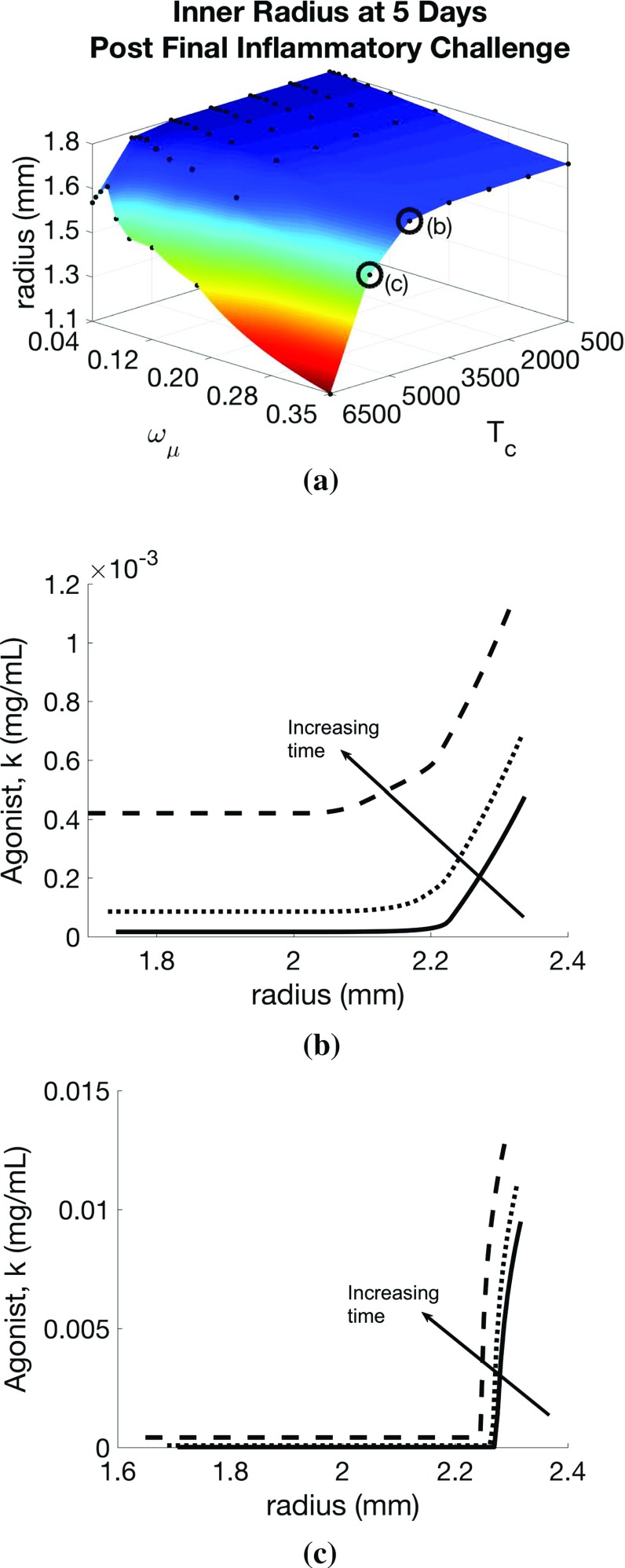
Effect of airway smooth muscle cell hyper-responsiveness on airway remodelling, active tone, and mechanotransductive feedback. Variation in **a** remodelled geometry with inflammation frequency ($\omega _\mu$) and hyper-responsiveness ($T_\mathrm{c}$) parameter values. Transmural contractile agonist concentration is plotted as a function of radius at days 28, 30, and 32 for parameter value pairs indicated by the circled points on **a**: **b** where contractile agonist eventually clears from the tissue and **c** where contractile agonist remains in the tissue in an indefinite feedback loop, causing increasing remodelling long after cessation of challenges

*Effect of changes in intrinsic ASM hyper-responsiveness* To simulate the effects of increasing intrinsic responsiveness of ASMCs to contractile agonist, we return to the inflammation-only challenges and investigate the effect of paired changes in responsiveness ($T_\mathrm{c}$) and challenge frequency ($\omega _{\mu }$). We find that remodelling increases with $T_\mathrm{c}$ and $\omega _{\mu }$ (Fig. 7a), but a threshold effect only exists for increasing $T_\mathrm{c}$. Increasing $T_\mathrm{c}$ also leads to much slower agonist clearance. At low values of $T_\mathrm{c}$, the agonist eventually clears (Fig. 7a, b), while at high values, a self-perpetuating feedback loop is established (Fig. 7a, c), due to the local mechanotransduction-driven agonist release, which is not resolved, and the airway eventually grows in to the lumen.

**Fig. 8:**
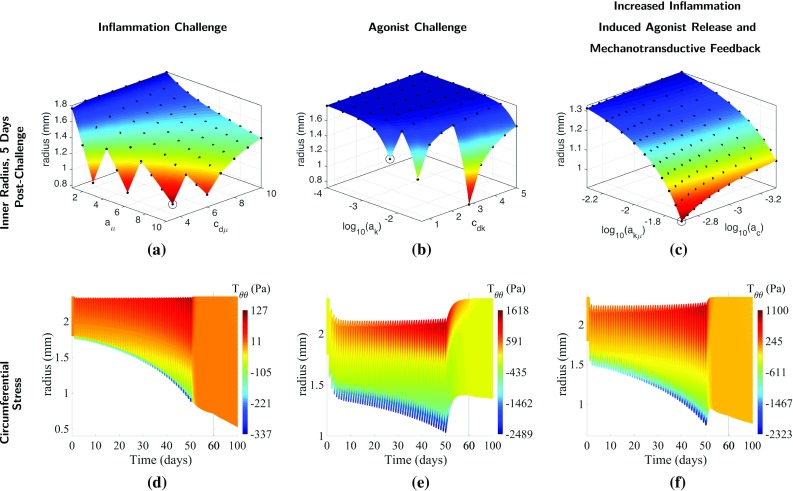
Global versus local effects on airway remodelling and active tone. Variation in remodelled geometry (1st row) with parameter pairs: inflammation magnitude and resolution ($c_{\mathrm{d}\mu }, a_\mu$; $1^{st}$ column), contractile agonist magnitude and resolution ($c_{\mathrm{d}k}, a_k$; $2^{nd}$ column), and inflammation-induced contractile agonist release and mechanotransductive agonist release ($a_{k\mu }, a_\mathrm{c}$; 3rd column). Circumferential stress (2nd row) is plotted as a function of radius and time for parameter value pairs indicated by the circled points on the surfaces

*Global versus local effects of inflammatory and contractile agonist challenges* In Figs. 4 and 6, we illustrate the effect of varying frequency and resolution rate of inflammation- and contractile agonist-only challenges, respectively. Here, we compare instead the effects of varying *amplitude* and resolution rate in these globally applied challenges (first and second columns, Fig. 8). Additionally, we compare these effects to changes in rates of inflammation- ($a_{k\mu }$) and stress-mediated ($a_\mathrm{c}$) local contractile agonist release (third column, Fig. 8).

Increasing the amplitude ($a _\mu$) and decreasing the resolution rate ($c_{\mathrm{d}\mu }$) of inflammatory-only challenges lead to increased remodelling (Fig. 8a), without a clear threshold effect. Additionally, the degree of remodelling becomes less dependent on the inflammation amplitude for sufficiently fast resolution. At the (relatively low) default value of ASMC responsiveness, $T_\mathrm{c}$, employed here, the contractile agonist clears rapidly from the tissue upon cessation of inflammatory challenges, due to the small amounts of local agonist activated by mechanical stress (Fig. 8d). Low levels of agonist release during inflammatory challenges results in limited contraction and therefore very low mechanical stresses.

In contrast, we observe a very sharp threshold for increasing contractile agonist magnitude ($a _k$) and decreasing agonist clearance rate ($c _{\mathrm{d}k}$) (Fig. 8b). Locally activated agonist remains in the tissue longer at lower clearance rates, and its effect is evident in the transmural variations in circumferential stress (Fig. 8f) in the time period following cessation of challenges (> day 50). High contractile agonist concentration induced by the agonist challenge generates significant bronchoconstriction and therefore relatively higher mechanical stresses than with inflammatory challenges.

Increasing the rate at which inflammation induces contractile agonist release ($a_{k\mu }$) exacerbates remodelling, and increasing the rate of stress-induced agonist release ($a_{\mathrm{c}}$) increases the positive mechanotransductive feedback, leading to additional remodelling (Fig. 8c). With both an increase in stress-mediated feedback and in inflammation-induced contractile agonist release, agonist resolution time is increased, though it remains significantly faster than with direct contractile agonist challenge. Increasing $a_{k\mu }$ and $a_{\mathrm{c}}$ thus leads to an effective combination of inflammatory and contractile agonist challenges, as increased contraction is observed during inflammatory challenges, and overall remodelling is higher than with agonist challenge alone. The higher levels of contractile agonist release during inflammatory challenges result in greater mechanical stresses (Figs. 8f) from agonist-induced bronchoconstriction.

**Fig. 9:**
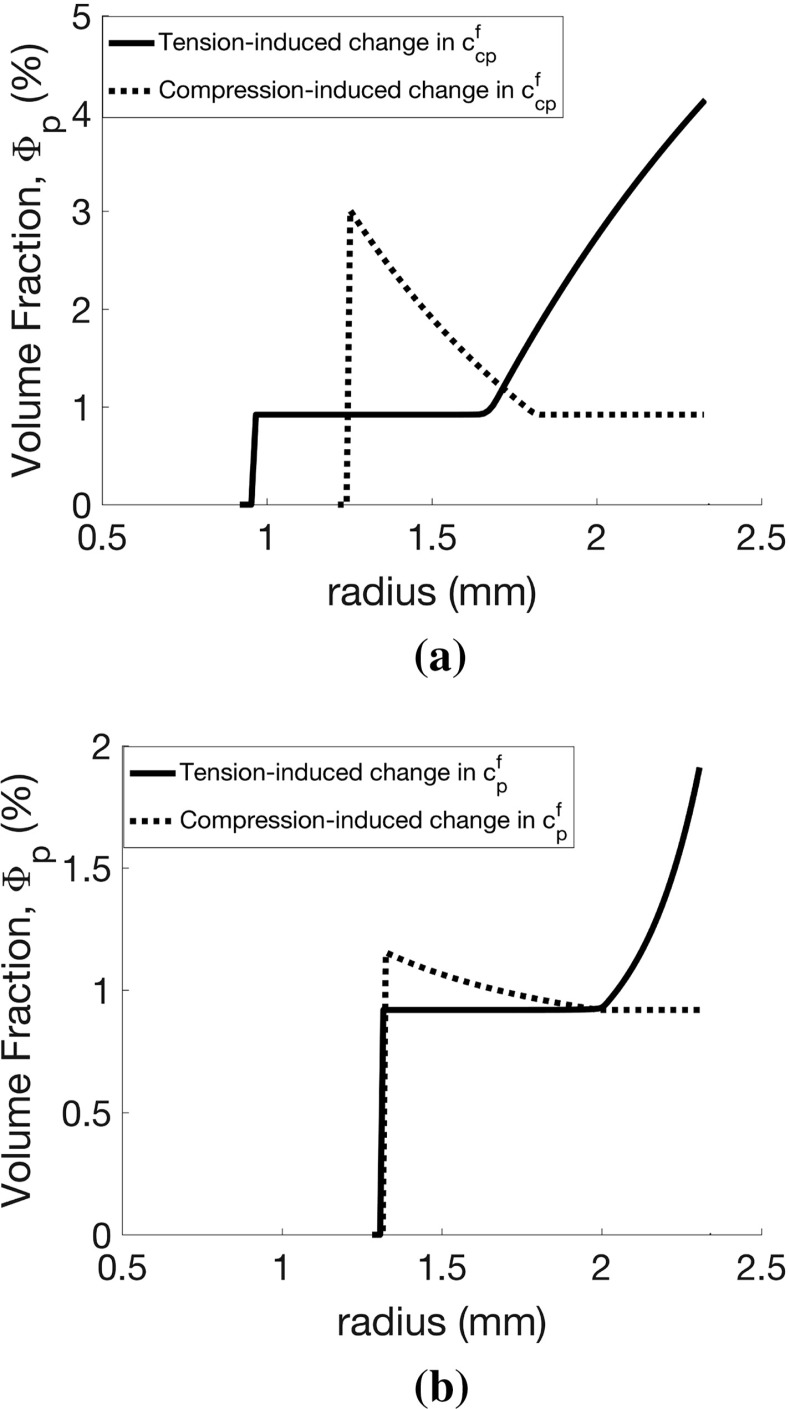
Effect of mechanotransductive mechanisms on airway mechanics and remodelling. Volume fraction of proliferating airway smooth muscle cells as a function of radius at day 51, for either tensile (solid lines) or compressive (dotted lines) mechanical stress-induced changes in ASMC (**a**) phenotype switching rate or **b** proliferation rate. Parameters for **a** are $c_{\mathrm{d}k} = 0.83$ and $c_\mathrm{cp}^f = 0.05$ and for **b** are $c_{\mathrm{d}k} = 0.50$ and $c_\mathrm{p}^f = 0.05$. Increased remodelling occurs in the former, even at a higher agonist clearance rate

*Effect of phenotype switching and proliferation rate modulation by tensile versus compressive mechanical stress* In all of the above simulations, we have assumed that only tensile stresses can increase phenotype switching (via (2.19) and nonzero $c_\mathrm{cp}^f$ in (2.20)) and that the proliferation rate of the proliferative ASMCs is unaffected by local stress (default value $c_\mathrm{p}^f =0$). Here, we investigate the effect of varying these parameters (in inflammatory-only challenges) for both tensile and compressive stress-modulated phenotype switching and proliferation/recruitment rate. We vary either the stress-induced switching rate ($c_\mathrm{cp}^f$) or the proliferation rate ($c_\mathrm{p}^f$) and the contractile agonist clearance rate ($c_{\mathrm{d}k}$). Transmural distributions of proliferating ASMC volume fraction at day 51 (Fig. 9), selected from the overall results of this parameter exploration (Online Resource 6), illustrate the effects of these parameters on remodelling.

We observe similar amounts of remodelling for increases in both tensile and compressive stress-modulated phenotype switching rate and decreasing agonist clearance rate with no clear threshold effect (see Online Resource 6). For the same parameter ranges, agonist resolution times are observed to be similar and relatively independent of $c_\mathrm{cp}^f$ for both cases. Distributions of the proliferative ASMC volume fraction significantly differ in the two cases, with larger volume fractions at the outer edge of the airway wall in the tensile stress-modulated case and at the inner edge in the compressive stress-modulated case (Fig. 9a), reflecting the observed circumferential stress heterogeneity and contractile agonist build-up. For our given initial conditions (Online Resource 2), both tensile and compressive stress-induced increase in phenotype switching results in a greater amount of airway remodelling than stress-induced increase in proliferation rate (Fig. 9a, cf. b). Agonist retention is similar between the two cases.

## Discussion

The mechanisms underlying the interaction of inflammation, airway hyper-responsiveness and airway remodelling in asthma are poorly understood. Thus, we have developed a new computational model of coupled biochemical- and mechanotransduction-induced remodelling of the airway wall. In the model, accumulation of ASM and ECM is driven by switching of ASMCs from a contractile to proliferative phenotype (which is capable of synthesizing ECM), and also, via a novel mechanotransductive feedback mechanism by which a mechanically stressed tissue releases mitogenic growth factors and contractile agonists.

Our results qualitatively match those reported for asthmatic airways (as well as results from our previous model; see Online Resource 7). For example, we have predicted narrowing of the lumen and a downward shift in the pressure–radius curves that have been previously reported in humans (Williamson et al. 2011). Our model also predicts wall thickening of airways due to increased ASM volume, which has been reported both in asthmatic patients (James et al. 2012, 2009) and in animal models (Alrifai et al. 2014).

The inflammatory twitch hypothesis suggests that self-limiting inflammatory events are invoked in the presence of an allergic stimulus and dissipate as the stimulus disappears, with long resolution periods being a possible explanation for the chronic inflammation characteristic of asthma (Pothen et al. 2016). Our results suggest that these long resolution periods may increase remodelling associated with severe asthma. In animal models of asthma, for example, transitory inflammatory cell recruitment, and increases in thickness of both ASM bundles and the SBM, have been associated with repeated/successive allergen challenges (Johnson et al. 2004; McMillan and Lloyd 2004). This response is thought to be the effect of pro-remodelling growth factors affecting activation of mast cells, ASM proliferation and ECM deposition (Naveed et al. 2017; James 2017) or increased recruitment of ASM progenitors such as myofibroblasts into the ASM bundles (Gerarduzzi and Battista 2017; Singh et al. 2014; Saunders et al. 2009). Thicknesses of both ASM bundles and the SBM have been shown to gradually return to control levels upon cessation of challenges (Alrifai et al. 2014; Leclere et al. 2012), with the resolution periods of inflammation and tissue growth being on the order of days to months (Pothen et al. 2016; Alrifai et al. 2014), which is reflected in our simulations, in which inflammatory status, $\mu$, remains elevated with decreased clearance rate, $c_{\mathrm{d}\mu }$.

Our work broadly follows that of Skalak (1980) and Rodriguez et al. (1994) in which growth and remodelling is treated as a topological mapping from a reference configuration to a grown configuration followed by an elastic deformation. A key component of this model is the implicit separation of timescales between the growth and the (instantaneous) elastic deformations. This approach has previously been applied to model airway narrowing (in which the folding that occurs during growth was modelled utilizing buckling theory; Moulton and Goriely 2011a, b), but this was in the context of a single phase, and growth kinematics were prescribed directly. Multiphase or mixture theory has been utilized in numerous cardiovascular studies (e.g. Valentin et al. 2013; Gleason and Humphrey 2005; Gleason et al. 2004; Humphrey and Rajagopal 2003, 2002), where the models were driven by evolution of the growth configuration of each phase, but without consideration of their interactions. To our knowledge, the approach taken here, in which the spatio-temporal evolution of the individual tissue constituents is driven by underlying biological and mechanochemical processes, has not been considered in airway remodelling. Thus, the multiphase model proposed here addresses the limitations of previous models by considering in detail the interactions between tissue constituents, as in the studies of Ateshian (2007) and Aparício et al. (2016). We use our model to carry out a series of parameter exploration studies to identify potential mechanisms underlying the pathogenesis and evolution of asthma, described below.

*Impaired resolution of inflammation may explain airway remodelling characteristic of asthma* As in our previous model (Chernyavsky et al. 2014), slower resolution of inflammation has a greater effect on airway wall thickening than either challenge frequency or amplitude (Figs. 4a, 8a), as a result of slower clearance of residual pro-remodelling and pro-contractile cytokines (See Online Resource 7). This effect may be responsible for increased remodelling and bronchoconstriction observed in patients with severe asthma that is poorly controlled with anti-inflammatory medications, e.g. corticosteroids. In these cases, inflammation may not be effectively cleared, e.g. by failure to induce inflammatory cell apoptosis (Wenzel 2012; Woolley et al. 1996).

*Inflammation-independent bronchoconstriction-mediated mechanical stresses could drive airway remodelling*Grainge et al. (2011) have demonstrated the possibility of inflammation-independent airway remodelling in asthmatics through application of methacholine-only challenges. Our model suggests the mechanisms by which this may occur. We show how tensile stresses along (circumferentially-oriented) muscle fibres, arising from agonist-induced contraction of ASMCs, can cause a local release of additional agonist (Fig. 6c). This mechanotransductive pathway may represent stretch-induced activation of latent TGF-$\beta$, which has both mitogenic (Halwani et al. 2011) and contractile agonist (Ojiaku et al. 2017; Oenema et al. 2013; Desmoulière et al. 2005; Grinnell and Ho 2002; Montesano and Orci 1988) properties. This additional local increase in agonist concentration triggers a local tensile stress-induced cell phenotype switching, thus driving increases in ASM mass. We have also shown that compressive stress-induced increases in phenotype switching or proliferation rate (Fig. 9), possibly mediated by shedding of growth factors such as EGFR and ET-1, could also explain the inflammation-independent remodelling observed in Grainge et al. (2011). Contractile agonists alone are insufficient to induce physiological changes in mice, as measured with plethysmography (Mailhot-Larouche et al. 2018). Our simulations suggest that very frequent challenges, impaired agonist clearance or increased rate of stress-driven agonist activation is required to cause significant remodelling (Fig. 8b). It is possible that an intrinsic inability to clear agonist, increased sensitivity of ASM/ECM to stress-driven TGF-$\beta$ activation or EGFR/ET-1 shedding could place the asthmatic subject in a high-risk region of the parameter space.

*Interaction of inflammation with intrinsic ASM hyper-responsiveness could explain persistent contractile tone and severe remodelling* Our simulations show that increased intrinsic hyper-responsiveness causes increased remodelling (Fig. 7a) and significantly increased contractile agonist resolution times. This response is a result of increased bronchoconstriction at a given agonist concentration, driving increases in tensile stresses and hence further (mechanotransductive) activation of pro-mitogenic mediators and contractile agonists such as TGF-$\beta$. These results highlight that the retention of contractile agonist associated with increased hyper-responsiveness is a candidate mechanism accounting for the persistent tone observed in asthmatics (Brightling et al. 2002). As expected, long resolution times are associated with impaired clearance of contractile agonist, $c_{\mathrm{d}k}$ (Fig. 6a). Notably, delayed contractile agonist resolution occurs when both the ASMCs are very hyper-responsive and inflammation challenge frequency is high. The combination of identified mechanisms could therefore be responsible for persistent contractile tone and remodelling, post-challenge, in hyper-responsive airways (Kariyawasam et al. 2007).

*Increased airway remodelling could occur via local mechanotransductive effects* An increase in both inflammation- and mechanical stress-induced contractile agonist release (Fig. 8c) leads to a condition that is an effective combination of inflammation-only (Fig. 8a) and agonist-only challenge (Fig. 8b). Increased rates of inflammation-induced agonist release ($a_{k\mu }$) could represent degranulation of the larger numbers of mast cells present in the ASM bundles of asthmatics (Naveed et al. 2017) and hence the production of contractile agonist (e.g. histamine). These released factors ultimately elicit a pro-mitogenic response where (tensile) mechanical stresses arising from ASMC contraction drive tissue remodelling via stress-induced phenotype switching of ASMCs. Separately, under the assumption of mechanically activated mediators (e.g. TGF-$\beta$), increasing stress-activated release ($a_\mathrm{c}$) increases agonist-induced bronchoconstriction, leading to further agonist release and thus further contraction and stress-induced remodelling in a perpetual feedback loop. The feedback mechanism may explain how coupled inflammation and bronchoconstriction lead to increased remodelling in severe asthma.

*Airway wall thickening may be a normal, local mechano-protective response but ultimately becomes detrimental to global lung mechanics and function* The increase in cross-sectional area from thickening of the SBM counter-intuitively may serve to reduce mechanical stresses, arising during bronchoconstriction (Fig. 5c). This reduction in stress could, in turn, reduce compressive stress-driven shedding of growth factors mediated by epithelial cells. Our model does not allow for buckling of the SBM, due to our imposition of axisymmetry, but reduced compressive stresses as a result of the thicker SBM could also reduce propensity to buckle (Moulton and Goriely 2011a) and hence limit airway narrowing during bronchoconstriction.

Our results also indicate that remodelling-induced changes in functional mechanical properties of the airways may depend on what drove the remodelling. For instance, we show that the pressure–radius curves during inflammation-only challenges have qualitatively different characteristics to those associated with agonist-only challenges (cf. Fig. 4b, 6b).

*Interaction of different underlying mechanisms may contribute to the existence of different asthma phenotypes* A combination of various characteristics defines asthma subgroups, or phenotypes (see Wenzel 2012 and references therein). Here, we have identified various parameter combinations that may contribute to the existence of different asthma phenotypes. For example, the presence of eosinophils in the lung tissue is associated with thickened SBM, high expression of TGB-$\beta$, increased frequency and severity of symptoms, and more near-fatal events (Wenzel 2012; Miranda et al. 2004). Persistent eosinophils, despite corticosteriod treatment (typically associated with eosinophil apoptosis), is associated with adult-onset, less allergic severe (T$_H$2) asthma (Wenzel 2012). The slower clearance of inflammation leading to increased remodelling (Figs. 4a, 8a) and reduced contractile agonist clearance may underlie this type of asthma. Our results (Fig. 5) suggest that a thickened SBM may be associated with the increased presence of eosinophils, in agreement with the SBM thickness observed in non-eosinophilic compared with eosinophilic asthma (Miranda et al. 2004).

Our results show that for regular bronchoconstrictive events to induce long-term airway remodelling an extreme set of consequences (Figs. 6, 8b) is required, which may be the reason why, to our knowledge, contractile agonist-induced remodelling has not been observed in animal studies (e.g. Mailhot-Larouche et al. 2018). However, the study by Grainge et al. (2011) shows that contractile agonist challenges do lead to increased ASM thickness in humans. That mice do not spontaneously develop asthma but that humans do, suggests that the subjects of the Grainge et al. (2011) study may lie in a different region of parameter space, i.e. beyond the relevant thresholds.

Methacholine challenges have been shown to increase airway responsiveness in humans over the short term (Gazzola et al. 2017), and increased smooth muscle responsiveness may be independent of inflammation in some asthma phenotypes (e.g. non-T$_H$2 asthma; Wenzel 2012). Our model suggests a possible mechanism: with intrinsically hyper-responsive ASMCs, normal exposure to contractile agonists alone (in the absence of inflammation) may be sufficient to drive further agonist release (Fig. 6b), and increase ASMC responsiveness, thus leading an asthma symptom phenotypes not typically associated with inflammation, such as exercise-, obesity-induced asthma, or non-T$_H$2 asthma.

Increased ASMC hyper-responsiveness (Fig. 7) and/or increased mechanotransduction (Fig. 8c) may exacerbate remodelling, even at normal levels of inflammation (pauci-granulocytic asthma; Carr et al. 2017). Thus, these mechanisms may underlie the degrees of severity associated with inflammation-induced (T$_H$2) asthma: mild to moderate asthmatics may be characterized by increased inflammation but normal ASMC responsiveness (Fig. 4), while severe asthmatics may have increased hyper-responsiveness that leads to pathological remodelling (Fig. 7). Alternatively, mechanisms that reverse influx of inflammatory cells, and hence, return inflammation to normal levels, may have become impaired (Pothen et al. 2016; Brightling et al. 2012). A remodelled airway exhibiting a combination of the characteristics described above may be primed for severe or even fatal bronchospasms in which airway contraction completely obstructs airflow.

*Model limitations and future work* Models of this nature necessarily require some simplifying assumptions. In particular, we have assumed that the airway remains axisymmetric (and free of shear) during both growth and elastic deformation. This simplified approach, in which only radial growth is considered, permits more straightforward analysis of the effects of varying concentrations of tissue constituents and contractile agonists across the airway wall. Nevertheless, our model is novel in accounting for local mechanotransductive effects and these spatial distributions. However, we note that some growth may occur circumferentially and, possibly, axially, in vivo. Circumferential growth/atrophy has been shown to underlie the development of residual stresses in arteries (Rodriguez et al. 1994); however, airways have been shown to exhibit little or no residual stresses (McKay et al. 2002), and so we omit this growth mode from the model for simplicity. Axial growth is similarly neglected, based on a lack of experimental data on axial growth of airways. The growth models of Ren (2013) and Grytsan et al. (2017) do account for both isotropic and varying degrees of anisotropic growth but do not consider heterogeneous spatial distributions. A more comprehensive approach would be to extend the models analysed herein to 3D. However, the numerical solution of the resulting equations is beyond the scope of the current study. Additionally, our imposition of axisymmetry prevents us from considering buckling of the stiff SBM. Future work could involve utilizing this model to predict regions of high compressive stresses in which buckling could occur (Moulton and Goriely 2011a).

To solve (2.27), we specify zero radial velocity at the outer wall, so that growth occurs inwardly. We note that this choice is somewhat arbitrary and that the growth velocity could be specified at any point along the wall thickness. We choose to specify zero growth on the outer boundary based on evidence that airways appear to narrow during asthma. Studies on human bronchial segments have shown that increased ASM in asthmatics contributes to exaggerated airway narrowing (Noble et al. 2013).

We do not allow for the separate evolution of the individual constituents’ reference configurations, as in Humphrey and Rajagopal (2002). If our assumptions were relaxed so that these could move independently, interphase drag may be used to more accurately account for stress-induced activation of latent TGF-$\beta$. Moreover, changes in reference configurations, in addition to non-axisymmetric growth, could allow for the development of residual stresses. Although healthy airways exhibit negligible or small residual stresses (McKay et al. 2002), to our knowledge these have not been measured in remodelled airways. Relaxing this constraint would present a challenge in the theoretical development and in the numerical solution that is beyond the scope of the current work.

We have assumed that collagen fibre recruitment initiates at stretches above unity, with an exponential function representing gradually increasing fibre uncrimping. Rigorous experimental analysis of the airway structure–function relationship, through coupled mechanical testing and advanced microscopic imaging (e.g. Hill et al. 2012; Clifford et al. 2011), would enable incorporation of fibre dispersion and recruitment to refine our model (e.g. Gasser et al. 2006; Sacks 2003; Lanir 1983, 1979). Also, we have not accounted for diffusion of applied contractile agonists, or of activated cytokines such as TGF-$\beta$. Finally, migration of myofibroblasts (ASM progenitors) into the muscle bundle has been hypothesized as a mechanism of increased ASM. Although this migration (and differentiation into ASMCs) has not been modelled here explicitly, the ASMC proliferation in our model could instead represent this recruitment of ASM progenitors, and the timescale associated with migration is implicitly accounted for in the proliferation rate (reinterpreted as a ‘recruitment’ rate of myofibroblasts which then become ASM).

Notwithstanding these limitations, key advantages of our model are the ability to generate measurable biological and mechanical output that may be tested experimentally and the ability to separate the long-term effects of growth from the relatively shorter term mechanical effects of pressurization and active contraction. A study, combining the modelling approach described here with experimental measurements of tissue constitution, geometry, and active mechanics, is required to distinguish between the effects of geometric remodelling and changes in tissue mechanics.

Therefore, this work forms the theoretical basis of a model that is currently being tested against data from in vivo animal experiments, utilizing an ovalbumin model of inflammation-induced asthma in mice. We have shown how the volume fraction of constituents may be evaluated at any time along the remodelling process (e.g. Fig. 4). These will be compared to measurements made on histological sections taken from animal models of asthma, to infer underlying mechanisms as discussed above, and also for further model development and validation. Additionally, we have demonstrated the ability to assess mechanical properties of the airway as they evolve, via computed pressure-radius curves (e.g. Fig. 4b) which are commonly measured experimentally, to show changes in passive and active tissue mechanics and distinguish between healthy and diseased airways.

In addition to airway remodelling, the approach outlined in this paper has broad applicability to other areas of tissue mechanics. For example, the mechanotransductive feedback mechanisms described herein likely have direct applications to models of aneurysm growth (Grytsan et al. 2017; Aparício et al. 2016). Moreover, the coupled biochemical–biomechanical elements are relevant to inflammation-associated adventitial collagen deposition observed in arteries under hypertension (Bersi et al. 2016). Furthermore, the technique we present for modelling a non-homogeneous spatial distribution of tissue constituents would be applicable to the myocardium, since local changes in tissue constitution occur during mechanical overload, e.g. in hypertension-induced myocyte hypertrophy and collagen deposition (Hill et al. 2014) and during remodelling following myocardial infarction (Gajarsa and Kloner 2011).

*Conclusions* Our results suggest that mechanical stresses, arising from bronchoconstriction, initiated by multiple inflammatory or contractile agonist challenges, and driving agonist release, generate a mechanotransductive feedback loop. With this feedback loop, increased ASM hyper-responsiveness to contractile agonists and impaired clearance of inflammatory factors and contractile agonists leads to increased remodelling, increased bronchoconstriction, and maintenance of an increased baseline contractile tone due to the chronic presence of contractile agonists. The key factors that allow such a state to emerge are (i) increased intrinsic hyper-responsiveness of ASMCs to contractile agonists, (ii) delayed contractile agonist clearance from the tissue, (iii) increased release of contractile agonist by inflammatory cells, and (iv) mechanotransductive feedback, in which active contraction generates increases in mechanical stress that subsequently initiate the release of additional contractile agonists and thus exacerbate remodelling. Targeting mechanotransductive pathways (e.g. TGF-$\beta$) and rapid resolution of inflammation or contractile agonist may have potential for treating severe forms of asthma.

## Electronic supplementary material

Below is the link to the electronic supplementary material.

Supplementary material 1 (pdf 23338 KB)
