# Supplementary material for: A theoretical model of inflammation- and mechanotransduction-driven asthmatic airway remodelling
Source: Biomech Model Mechanobiol. 2018 Jul 2;17(5):1451–70. doi: 10.1007/s10237-018-1037-4 (PMC6154265; doi:10.1007/s10237-018-1037-4)
Supplement: Supplementary file 1 — Supplementary material 1 (pdf 23338 KB) [file 10237_2018_1037_MOESM1_ESM.pdf]

Online Resources: A theoretical model of inflammation- and mechanotransduction-driven asthmatic airway remodelling

1 Additional Mathematical Details

This section provides additional details about the model development and numerical solution techniques.

1.1 Fiber Directions

Considering a cylindrical body composed of an anisotropic material reinforced by two sets of fibers dispersed in the $\theta - z$ plane, the undeformed fibre directions in polar cylindrical co-ordinates are given by

$$\mathbf{m}_{0,a}^{(1)} = \cos\Theta_a \mathbf{e}_\theta + \sin\Theta_a \mathbf{e}_z, \tag{S1a}$$

$$\mathbf{m}_{0,a}^{(2)} = -\cos\Theta_a \mathbf{e}_\theta + \sin\Theta_a \mathbf{e}_z, \tag{S1b}$$

for a given constituent a, where $\mathbf{e}_\theta$ and $\mathbf{e}_z$ are unit vectors in the circumferential and axial directions, respectively. The current fibre direction, denoted $\mathbf{m}_a^{(j)}$, is obtained from the undeformed fibre directions via a push-forward operation,

$$\mathbf{m}_a^{(j)} = \frac{\mathbf{F}\cdot\mathbf{m}_{0,a}^{(j)}}{\sqrt{\alpha_a^{(j)}}}, \tag{S2a}$$

where

$$\alpha_a^{(j)} = \mathbf{m}_{0,a}^{(j)} \cdot \mathbf{C}\mathbf{m}_{0,a}^{(j)}, \qquad j = 1, 2, \tag{S2b}$$

is the square of the fibre stretch ratio (Holzapfel, 2000), and $\mathbf{C} = \mathbf{F}^T\mathbf{F}$ is the right Cauchy-Green tensor. We further posit that all fibres, including both ASM and collagen fibres in the ECM, are oriented along the circumference (Ijpma et al, 2017), so $\Theta_a = \Theta = 0°$.

1.2 Specific Forms of the Strain Energy Functions

Below we define the forms of the strain energy functions for the tissue constituents $a = p, c, e$. The neo-Hookean form of the strain energy function for the proliferating (p) airway smooth muscle cells (ASMCs) is given by

$$W_p = \frac{1}{2}\eta_p\left(I_1 - 3\right), \tag{S3a}$$

where η_p is a material parameter representing the passive stiffness of proliferating cells, and $I_1 = \mathrm{tr}\mathbf{C}$ is a strain invariant. The form for the contractile (c) ASMCs is given by

$$W_c = \frac{1}{2}\eta_c\left(I_1 - 3\right) + \sum_{j=1,2}\frac{C_c}{2\beta_c}\left(\exp^{\beta_c\left(\alpha_c^{(j)}-1\right)^2} - 1\right), \tag{S3b}$$

where material parameters are η_c, representing the passive (isotropic) stiffness of contractile cells, C_c, representing their passive (anisotropic) stiffness, and β_c, accounting for nonlinear stiffening with increasing deformation. The form for the extracellular matrix (ECM or e) is modeled similarly, so

$$W_e = \frac{1}{2}\eta_e\left(I_1 - 3\right) + \sum_{j=1,2} H\left(\alpha_e^{(j)} - {\lambda_u}^2\right)\frac{C_e}{2\beta_e}\left(\exp^{\beta_e\left(\alpha_e^{(j)}-{\lambda_u}^2\right)^2} - 1\right), \tag{S3c}$$

with material parameters η_e, representing the passive (isotropic) stiffness of the embedded ECM cells, C_e, representing fibre density, and β_e, parametrizing the gradual recruitment of collagen fibres.

1.3 Numerical Solution Procedure

This section provides specific forms of the model equations that were used in the numerical scheme, as well as the numerical techniques that were used to solve the PDEs. First, we derive the equations governing the growth in each layer of the airway wall. Next, we derive a nonlinear equation that represents the elastic deformation of a two-layer multi-phase cylinder subject to pressure boundary conditions. Finally, we discuss the specific numerical schemes we used to solve these equations and the governing PDEs in the main text.

Growth. The radial growth of the airway is determined as follows. Integrating (2.27) with respect to ξ gives the velocity in the outer layer as

$$\xi v(\xi)^{(o)} = \int_{\xi_{int}}^{\xi} \xi' q^{(o)} d\xi' + K_1, \qquad \xi_{int} \le \xi \le \xi_2. \tag{S4a}$$

Similarly, the velocity in the inner layer is given by

$$\xi v(\xi)^{(i)} = \int_{\xi_1}^{\xi} \xi' q^{(i)} d\xi' + K_2, \qquad \xi_1 \le \xi \le \xi_{int}. \tag{S4b}$$

The constants K_1 and K_2 are determined by applying the zero velocity boundary condition (2.31c) and continuity of velocity at ξ_{int} (2.31d), respectively. The interface velocity is then given by (S4a) evaluated at $\xi = \xi_{int}$ to give

$$v(\xi_{int}) = \frac{d\xi_{int}}{dt} = -\frac{1}{\xi_{int}} \int_{\xi_{int}}^{R_2} \xi q^{(o)} d\xi, \tag{S4c}$$

which is solved numerically for ξ_{int}. It is then used with (S4b) to obtain an expression for the velocity at the inner wall, given by

$$v(\xi_1) = \frac{d\xi_1}{dt} = \frac{1}{\xi_1} \left[\xi_{int} v(\xi_{int}) - \int_{\xi_1}^{\xi_{int}} \xi q^{(i)} d\xi \right], \tag{S4d}$$

which is again solved numerically for ξ_1.

Elastic deformation. Integrating (2.12) and applying (2.13a) gives the radial stress for the inner layer

$$T_{rr}^{(i)} = \int_{r_1}^{r} \frac{1}{r'} \left(T_{\theta\theta}^{(i)} - T_{rr}^{(i)} \right) dr' - P_1, \qquad r_1 \le r \le r_{int}, \tag{S5a}$$

and applying (2.13d) gives the radial stress for the outer layer

$$T_{rr}^{(o)} = T_{rr}^{(i)}(r_{int}) + \int_{r_{int}}^{r} \frac{1}{r'} \left(T_{\theta\theta}^{(o)} - T_{rr}^{(o)} \right) dr' - P_2, \qquad r_{int} \le r \le r_2. \tag{S5b}$$

Applying continuity of stress (2.13c) thus gives

$$P_1 - P_2 = \int_{r_1}^{r_{int}} \frac{1}{r} \left(T_{\theta\theta}^{(i)} - T_{rr}^{(i)} \right) dr + \int_{r_{int}}^{r_2} \frac{1}{r} \left(T_{\theta\theta}^{(o)} - T_{rr}^{(o)} \right) dr, \tag{S5c}$$

wherein r_1 and r_2 can be expressed in terms of r_{int} via (2.4), since ξ_1 and ξ_{int} are known from the solution of (S4), and (i) denotes variables computed in the inner layer and (o) those in the outer layer. Together with the radial and circumferential stress components of the Cauchy stress specified by (2.5) and (S3), (S5c) is therefore an algebraic equation in the unknown r_{int}. At each time step, a root finding algorithm (`fzero.m`), is used to solve the equilibrium equation (S5c) for r_{int}. All other variables can be evaluated once this is known.

Table 1: Initial Conditions and Airway Geometry

Parameter	Definition	Inner layer	Outer layer	Units
$\mu\|_{t_0}$	Inflammatory factor, μ	0	0	mg mm^{-3}
$k\|_{t_0}$	Contractile agonist, k	0	0	mg mm^{-3}
$\Phi_c\|_{t_0}$	Contractile ASMC volume fraction	0	0.20	
$\Phi_p\|_{t_0}$	Proliferating ASMC volume fraction	0	1.50x10^{-3}	
$\Phi_e\|_{t_0}$	ECM volume fraction	0.30	9.85x10^{-2}	
R_1	Inner radius	1.800		mm
R_{int}	Interface radius	1.818		mm
R_2	Outer radius	2.340		mm

Numerical techniques. Numerical solutions to the system of coupled PDEs, given by (2.26), with (2.18-2.25), were obtained via the method of lines as follows. A finite difference spatial discretisation, with upwinding applied to convective terms, was employed. For simplicity, we fixed with the number of nodes at 10 in the (thin) inner layer and 100 in the outer layer, noting that, as the airway grows, Δr is not constant[1]. The resulting system, along with (S4), was time-stepped in MATLAB using an ODE solver (`ode45.m` or `ode15s.m`; the latter is used when inflammatory or agonist challenge frequency is very high resulting in a stiff system of equations), with the integrals evaluated using `trapz.m`. This method was applied separately to the inner and outer layers and solutions matched at r_{int}.

2 Model Parameters and Initial Conditions

Initial conditions for inflammatory factor, μ, contractile agonist concentration, k, and volume fractions, Φ_a, $a = p, c, e$, along with the initial geometry of the airway, are given in Table 1. Rate constants for the mass balance equations and material parameters, consistent between the two layers, are given in Table 2. Model parameters differing between the layers are given in Table 3.

3 Sensitivity Study

We performed a one-at-a-time sensitivity study by varying parameters a_μ, $c_{d\mu}$, a_k, c_{dk}, T_c, $a_{k\mu}$, and a_c (Fig. S1). For each parameter, simulations were performed for a range of 100 values, with inflammatory challenges (except for parameter a_k, in which the airways were challenged with contractile agonists) at a frequency of one per day for 50 days, followed by a resolution period. Change in inner radius, from the initial value (R_1=1.8mm), at 5 days post final challenge was used to assess the results. The model is highly sensitive to a_μ and $c_{d\mu}$, as increased remodelling (represented by decreased inner radius) is associated with increasing magnitude (a_μ), decreasing clearance ($c_{d\mu}$) of inflammatory factor μ, with the former exhibiting a linear response above a certain threshold and the latter a nonlinear response. Moreover, the model is highly sensitive to contractile agonist magnitude (a_k) in the agonist-challenge simulations. Note that the curve for a_k in Fig. S1 does not pass through zero. The reason for this is that, at the default value of a_k and c_{dk} (Table 2), the simulations with contractile agonist challenges at a frequency of 1 per day over 50 days results in contraction into the lumen. A nonlinear decrease in radius is associated with decreasing clearance of contractile agonist concentration, k, with a strong threshold effect observed with decreasing c_{dk}. The model is less sensitive to this parameter than magnitude, clearance of μ (subject to inflammatory challenges).

[1] Remark: Initially, $\Delta r = 0.002mm$ in the inner layer and $0.005mm$ in the outer layer. For all times, $\Delta r < 0.2mm$, even in the extreme (unrealised) case in which only the airway inner layer grows into the lumen.

Table 2: Default Model Parameters Consistent Between the Two Layers

Parameter	Definition	Value	Units
Constants			
ρ_c^T	Contractile ASMC density	1.050	mg mm^{-3}
ρ_p^T	Proliferative ASMC density	1.050	mg mm^{-3}
ρ_e^T	Extracellular matrix density	1.050	mg mm^{-3}
ρ_f^T	Fluid mass density	1.000	mg mm^{-3}
Φ_w	Fluid volume fraction	0.70	
Inflammation and Agonist Rate Constants			
a_μ	Stimulus amplitude for inflammatory factor, μ	3	mg mm^{-3} day^{-1}
$c_{d\mu}$	Decay rate of inflammatory factor, μ	5	day^{-1}
μ_1	First inflammatory threshold	1	mg mm^{-3}
μ_2	Second inflammatory threshold	2.5	mg mm^{-3}
a_k	Stimulus amplitude for contractile agonist, k	4.64x10^{-2}	mg mm^{-3} day^{-1}
c_{dk}	Decay rate of contractile agonist, k	2	day^{-1}
$a_{k\mu}$	Inflammation-induced agonist release coefficient	0.001	day^{-1}
a_c	Contraction-induced agonist release coefficient	0.001	mg mm^{-3} cmH$_2$O^{-1} day^{-1}
Event Parameters			
d	Duration of inflammation/agonist administration	1/3	
σ	Event parameter	0.01	
ω	Inflammation, μ, or contractile agonist, k, challenge frequency	1	day^{-1}
T	Time scale	1000	days
Mechanical/Material Parameters			
η_p	Proliferative ASMC passive isotropic stiffness	51.84	cmH$_2$O
η_c	Contractile ASMC passive isotropic stiffness	51.84	cmH$_2$O
C_c	Contractile ASMC passive anisotropic stiffness	1.14x10^{-3}	cmH$_2$O
β_c	Contractile ASMC passive anisotropic exponential parameter	2.74	
η_e	ECM passive isotropic stiffness	51.84	cmH$_2$O
C_e	ECM passive anisotropic stiffness	18.1	cmH$_2$O
β_e	ECM passive anisotropic exponential parameter	1.48	
T_c	Agonist-induced active contraction parameter	1000	cmH$_2$O
λ_{act}	Collagen recruitment stretch	1	
λ_z	Axial stretch ratio	1	
Θ	Fiber angle	0	radians
P_1	Lumen pressure	0	cmH$_2$O
P_2	External pressure	0	cmH$_2$O

Also, the model is not very sensitive to changes in ASMC responsiveness to contractile agonist (T_c), inflammation-induced contractile agonist release ($a_{k\mu}$), and mechanical-stress induced contractile agonist release (a_c), as each of these result in only small (nonlinear) changes in inner radius.

4 Volume Fractions

In order to compare results more directly from the simulations in Figs. 4 and 6, we plot the volume fractions of the airway wall constituents (proliferating, contractile ASMCs and ECM) as functions of the radius in Fig. S2. The left column depicts the constituent volume fractions taken at 3 separate days (increasing in time, moving down the column) from the simulation using the parameters corresponding to the circled point on the surfaces of Fig. 4a,b, while the right column depicts those corresponding to the circled point on the surfaces of Fig. 6a,b. The volume fractions of the constituents remain flat and only slightly increase during inflammation challenges (moving down

Table 3: Default Model Parameters Differing Between the Two Layers

Parameter	Definition	Inner layer	Outer layer	Units
c_{p0}	Baseline cell proliferation rate constant	0	1/3	day^{-1}
c_{pc}	Proliferative to contractile ASMC switching rate constant	0	2/3	day^{-1}
c_{c0}	Basline (low) contractile to proliferative switching rate constant	0	2.50x10^{-3}	day^{-1}
c_{c1}	Medium contractile to proliferative switching rate constant	0	5.0x10^{-3}	day^{-1}
c_{c2}	High contractile to proliferative switching rate constant	0	5.0x10^{-2}	day^{-1}
c_a	Contractile cell apoptosis rate constant	0	1.19x10^{-2}	day^{-1}
c_{de}	Baseline ECM degradation rate constant	0	9.70x10^{-3}	day^{-1}
c_{e0}	Baseline (low) ECM deposition rate constant	0	1.0x10^{-3}	mg mm^{-3} day^{-1}
c_{e1}	Medium ECM deposition rate constant	0	1.0x10^{-3}	mg mm^{-3} day^{-1}
c_{e2}	High ECM deposition rate constant	0	1.0x10^{-3}	mg mm^{-3} day^{-1}
c_{pe}	ECM deposition, via proliferative cells, rate constant	0	1.0x10^{-3}	day^{-1}
c_p^f	Stress-induced cell proliferation rate constant	0	0	day^{-1}
c_{cp}^f	Stress-induced contractile to proliferative ASMC switching rate constant	0	5.0x10^{-3}	day^{-1}

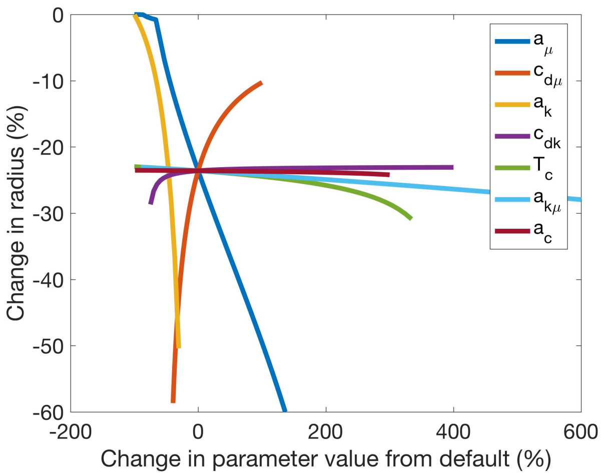

Fig. S1: *Sensitivity Study.* Change in inner radius, at 5 days post final challenge, from original radius R_1=1.8mm, as a function of change in parameter from default value (Table 2). The airway was challenged every day for a 50 day period with inflammation challenges, except for the study varying a_k, in which contractile agonist challenges were used. The default value for a_k was chosen so that low frequency challenges led to non-trivial remodelling, but at higher frequencies used here (one per day for 50 days), growth/contraction into the lumen results with this default value.

the left column). The increase in proliferating ASMCs towards the outer wall of the airway is due to the tensile mechanical stress-induced increase in phenotype switching rate. Thus, the figures in the right column depict the local increase in proliferative, and associated decrease in contractile, ASMCs during phenotype switching. Also, the airway geometry shifts to the right from day 28 to day 32, as the contractile agonist gradually clears from the tissue and the airway relaxes.

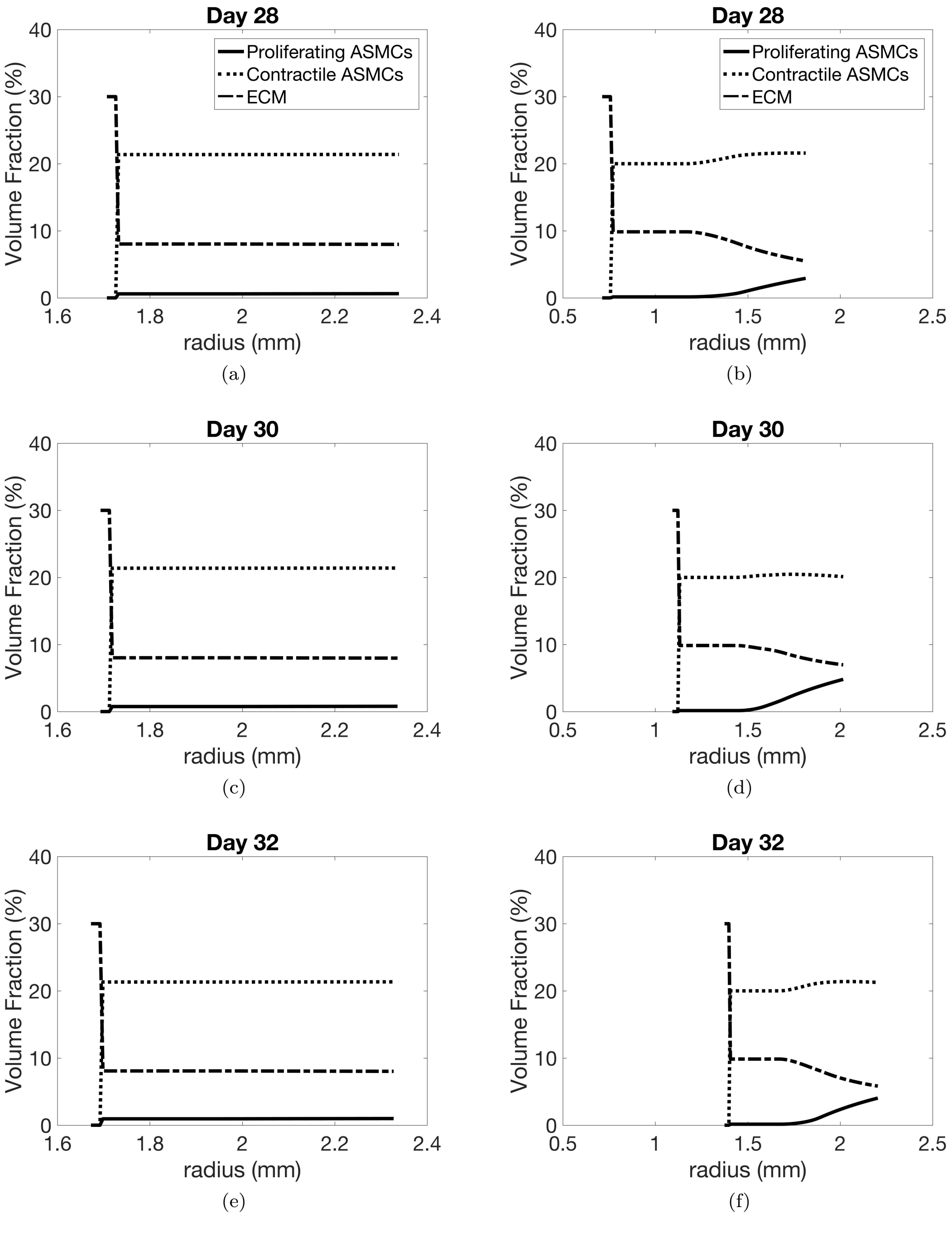

Fig. S2: *Volume Fractions vs. Radius.* Volume fractions of proliferating, contractile airway smooth muscle cells (ASMCs) and extracellular matrix (ECM) plotted as a function of radius taken at days 28, 30, and 32, corresponding to (left column) the circled point on the surfaces of Fig. 4a,b and (right column) the circled point on the surfaces of Fig. 6a,b. The inner radius shifts to the left more dramatically moving down the right column compared with the left column, as contractile agonist is cleared from the tissue following the challenge. Clearly, contractile agonist-induced deformation is dominant in the agonist-challenge simulations.

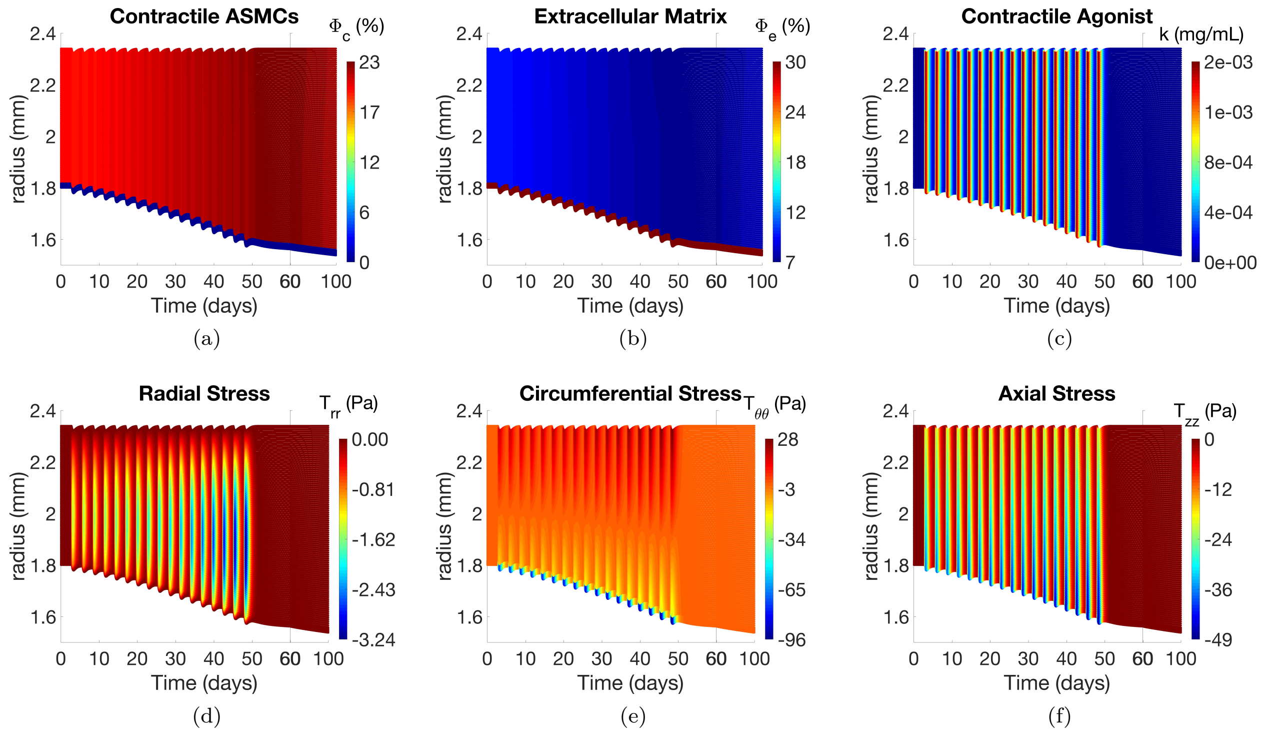

Fig. S3: *Volume Fractions, Contractile Agonist Concentration, and Mechanical Stresses during Inflammatory Challenges.* Illustrative results are evaluated at the circled point on the surface of Fig. 4a: volume fractions of (a) contractile ASMCs, (b) extracellular matrix, and concentration of (c) contractile agonist; Cauchy stresses in the (d) radial, (e) circumferential, and (f), (f) axial directions

5 Volume Fractions, Contractile Agonist Concentration, and Mechanical Stresses

The volume fractions, local contractile agonist concentrations, and mechanical stress distributions for the selected points (not already included) in Figs. 4 and 6 are depicted in Figs. S3 and S4, respectively. During inflammation-only challenges, the gradients of the constituents and agonists across the airway radius (Figs. S3a-c and 4c) are low compared to contractile agonist challenges (Figs. S4a-c and 6c), in which the local mechanotransduction-induced ASMC phenotype switching leads to local increases in ASM towards the outer wall (associated with regions on increased circumferential tensile stress), and thus relatively higher volume fractions of proliferating ASMCs and lower volume fractions of contractile ASMCs and ECM. For both inflammation (Fig. S3d-f) and agonist (Fig. S4d-f) challenges, radial stresses are compressive in the mid-wall and zero at the boundaries (thus matching the zero pressure boundary conditions), circumferential stresses are tensile in the outer potion of the wall and compressive in the inner portion, and axial stresses are compressive (due to incompressibility), with agonist challenges resulting in much higher stress magnitudes due to the active contraction.

6 Effect on Remodelling of Changes in Phenotype Switching Rate or Intrinsic Proliferation Rate Modulated by Mechanical Tensile or Compressive Stresses

Similar amounts of remodelling are observed for increases in both tensile and compressive stress-modulated phenotype switching rates, c_{cp}^{f} and decreasing agonist clearance rate, c_{dk}, with no clear threshold effect (Fig. S5a,b). For the same parameter ranges, agonist resolution times are also observed to be similar (note some simulation results are not plotted due to contraction/growth into the lumen during challenges). Moreover, agonist resolution time appears to be relatively independent of c_{cp}^{f} for both cases (Fig. S5c,d). For a selected parameter set (shown as circles on the surfaces in Figs. S5a–d), distributions of the proliferative ASMC volume fraction are significantly different in the two cases. Larger volume fractions are observed at the outer edge of the airway wall in the tensile stress-modulated case (Fig. S5e) and at the inner edge in the compressive stress-modulated case (Fig. S5f).

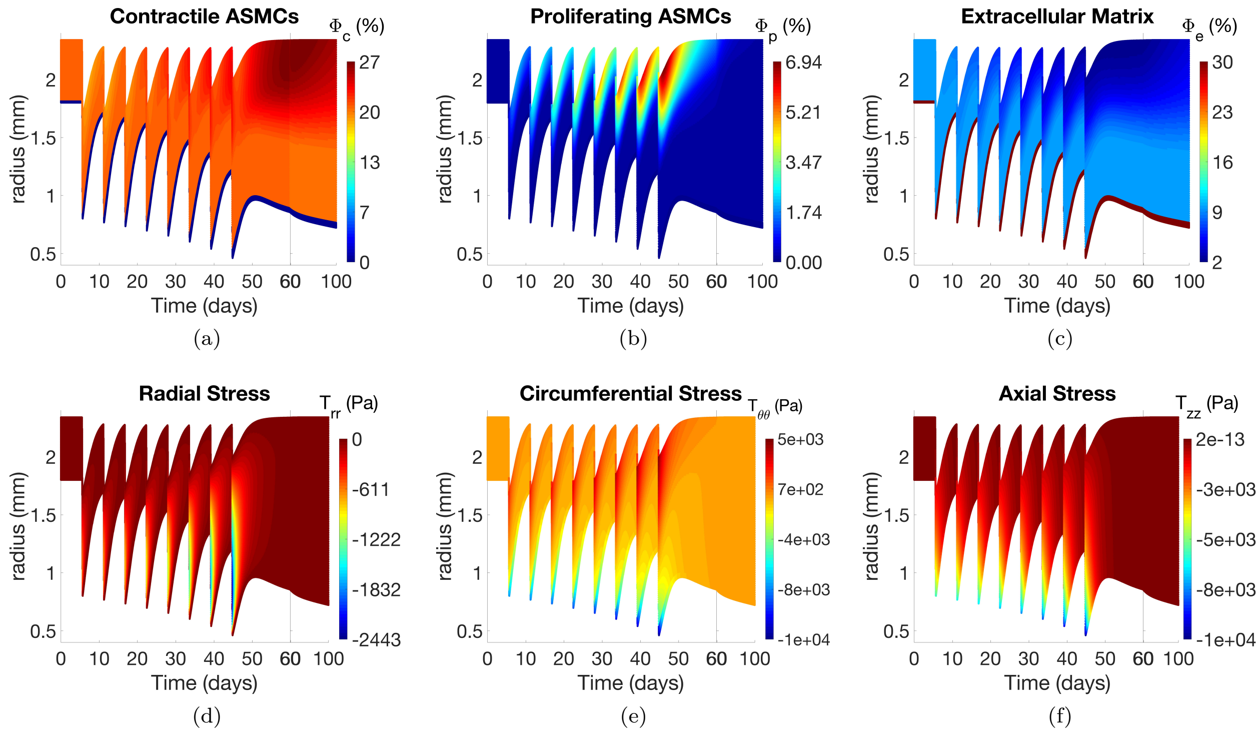

Fig. S4: *Volume Fractions, Contractile Agonist Concentration, and Mechanical Stresses during Contractile Agonist Challenges.* Illustrative results are evaluated at the circled point on the surface of Fig. 6a: volume fractions of (a) contractile, (b) proliferating ASMCs, and (c) extracellular matrix; Cauchy stresses in the (d) radial, (e) circumferential, and (f) axial directions

For our given initial conditions, both tensile and compressive stress-induced phenotype switching (c^f_{cp}) results in a greater amount of airway remodelling (Fig. S5a,b) than stress-induced increase in proliferation rate (c^f_p; Fig. S6a,b); again, note that some simulation results are not plotted due to contraction/growth into the lumen during challenges. Agonist retention is similar between the two cases (cf. Figs. S5c,d, S6c,d). Slightly less contraction is observed during challenges with increasing c^f_{cp} compared with increasing c^f_p. In the former case (Fig. S5e, S5f), contractile cells are lost due to phenotype switching, and in the latter case (Fig. S6e, S6f), the intrinsic proliferation rate of the current (lower) population of proliferating ASMCs is increased.

7 Comparison to Previous Modelling Results

Qualitatively, very similar results were obtained between the current study and our previous study (Chernyavsky et al (2014); Fig. S7). Severe remodelling in the former (red colour in Figs. S7a,b) corresponds to increased remodelling towards the lumen in the latter (red colour in Figs. S7c,d, respectively corresponding to the inward remodelling shown in Figs. 8a, 4a).

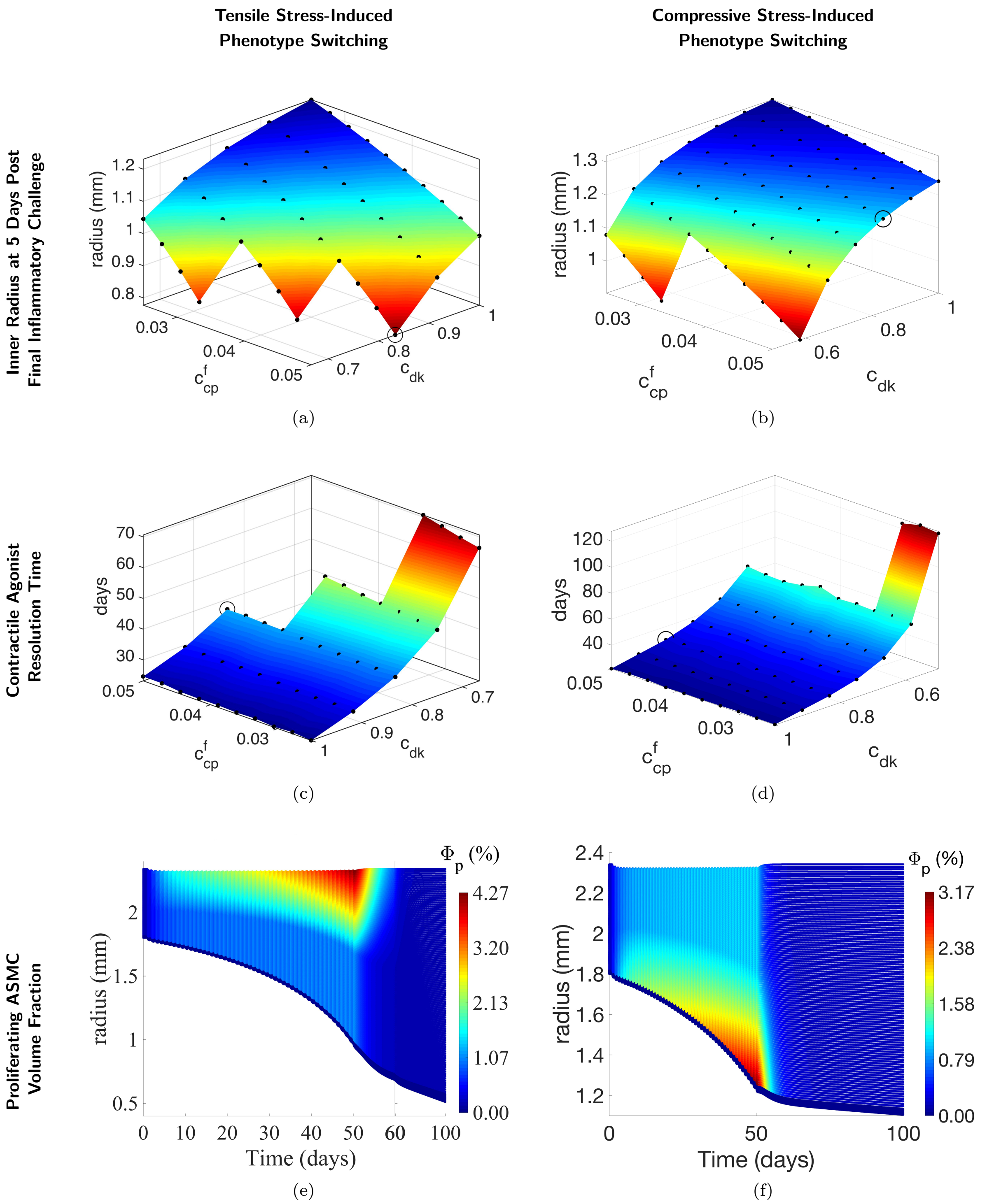

Fig. S5: *Effect of phenotype switching rate modulated by tensile vs compressive stress*: Variation in (a), (b) remodelled geometry (1st row) and (c), (d) agonist resolution rate (2nd row) with selected parameter values of stress-induced phenotype switching (c^f_{cp}) and agonist resolution rate (c_{dk}). The proliferating airway smooth muscle cell volume fraction (e), (f) is plotted as functions of radius and time for parameter value pairs indicated by the circled points on the surfaces.

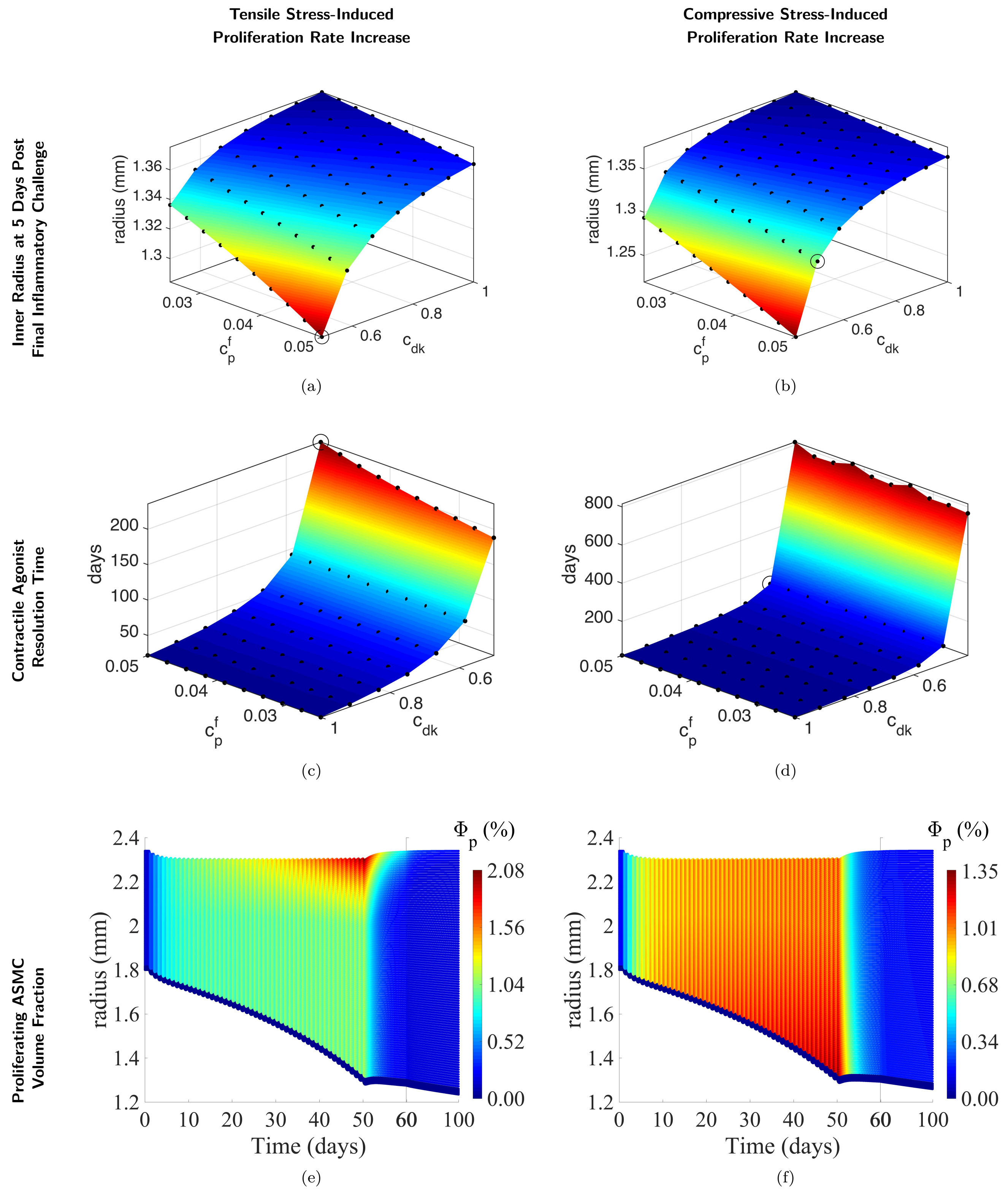

Fig. S6: *Effect of proliferation rate modulated by tensile vs compressive stress*: Variation in (a), (b) remodelled geometry (1st row) and (c), (d) agonist resolution rate (2nd row) with selected parameter values of stress-induced proliferation rate increase (c_p^f) and agonist resolution rate (c_{dk}). The proliferating airway smooth muscle cell volume fraction (e), (f) is plotted as functions of radius and time for parameter value pairs indicated by the circled points on the surfaces.

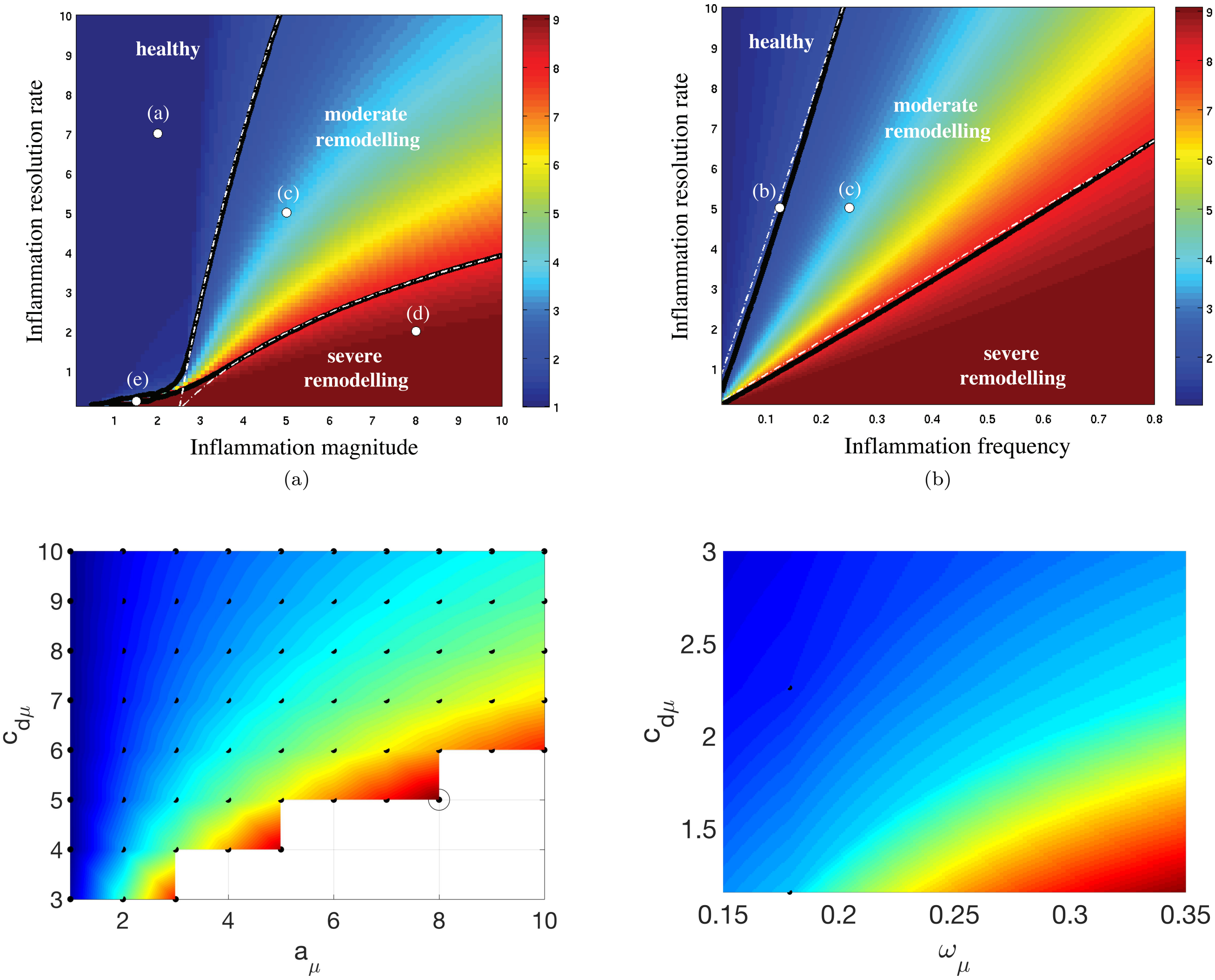

(a) (b) (c) (d)

Fig. S7: *Comparison of Current Model Results to Chernyavsky et al (2014).* Top row: results from Chernyavsky et al (2014), showing fold-increase in ASM population size after 300 days (colour scale) as a function of the inflammation resolution rate and the (a) inflammation magnitude or (b) inflammation challenge frequency; Bottom row: results from current study, showing inner radius at 5 days post final inflammatory challenge (colour scale) as a function of the inflammation resolution rate and the (c) inflammation magnitude (rotated view of Fig. 8a) or (d) challenge frequency (rotated and zoomed view of Fig. 4a).

References

Alrifai M, Marsh LM, Dicke T, Kilic A, Conrad MC, Renz H, Garn H (2014) Compartmental and temporal dynamics of chronic inflammation and airway remodelling in a chronic asthma mouse model. PLOS ONE 9(1):e85,839

Aparício P, Thompson MS, Watton PN (2016) A novel chemo-mechano-biological model of arterial tissue growth and remodelling. Journal of biomechanics 49(12):2321–2330

Ateshian G (2007) On the theory of reactive mixtures for modeling biological growth. Biomechanics and Modeling in Mechanobiology 6:423–445

Ateshian G, Ricken T (2010) Multigenerational interstitial growth of biological tissues. Biomechanics and Modeling in Mechanobiology 9:689–702

Ateshian GA (2011) The role of mass balance equations in growth mechanics illustrated in surface and volume dissolutions. Journal of Biomechanical Engineering 133(1):011,010

Benayoun L, Druilhe A, Dombret MC, Aubier M, Pretolani M (2003) Airway structural alterations selectively associated with severe asthma. American Journal of Respiratory and Critical Care Medicine 167(10):1360–1368

Berair R, Saunders R, Brightling CE (2013) Origins of increased airway smooth muscle mass in asthma. BMC medicine 11(1):145

Bersi MR, Bellini C, Wu J, Montaniel KR, Harrison DG, Humphrey JD (2016) Excessive adventitial remodeling leads to early aortic maladaptation in angiotensin-induced hypertension. Hypertension DOI 10.1161/HYPERTENSIONAHA.115.06262

Bossé Y, Chin LY, Paré PD, Seow CY (2009) Adaptation of airway smooth muscle to basal tone: relevance to airway hyperresponsiveness. American journal of respiratory cell and molecular biology 40(1):13–18

Bowen R (1976) Theory of Mixtures. In Continuum Physics, ed. AC Eringen. Academic Press, New York

Brightling C, Bradding P, Pavord I, Wardlaw A (2003) New insights into the role of the mast cell in asthma. Clinical & Experimental Allergy 33(5):550–556

Brightling C, Gupta S, Gonem S, Siddiqui S (2012) Lung damage and airway remodelling in severe asthma. Clinical & Experimental Allergy 42(5):638–649

Brightling CE, Bradding P, Symon FA, Holgate ST, Wardlaw AJ, Pavord ID (2002) Mast-cell infiltration of airway smooth muscle in asthma. New England Journal of Medicine 346(22):1699–1705

Brook B, Peel S, Hall I, Politi A, Sneyd J, Bai Y, Sanderson M, Jensen O (2010) A biomechanical model of agonist-inititated contraction in the asthmatic airway. Respiratory Physiology and Neurobiology 170:44–58

Brown RH, Togias A (2016) Measurement of intra-individual airway tone heterogeneity and its importance in asthma. Journal of Applied Physiology p 00545

Carr TF, Zeki AA, Kraft M (2017) Eosinophilic and non-eosinophilic asthma. American Journal of Respiratory and Critical Care Medicine DOI 10.1164/rccm.20161102232PP

Chernyavsky I, Crosier H, Chapman L, Kimpton L, Hiorns J, Brook B, Jensen O, Billington C, Hall I, Johnson S (2014) The role of inflammation resolution speed in airway smooth muscle mass accumulation in asthma: Insight from a theoretical model. PLOS ONE 9(3):e90,162

Clifford PS, Ella SR, Stupica AJ, Nourian Z, Li M, Martinez-Lemus LA, Dora KA, Yang Y, Davis MJ, Pohl U, Meininger GA, Hill MA (2011) Spatial distribution and mechanical function of elastin in resistance arteries. Arteriosclerosis, thrombosis, and vascular biology 31(12):2889–2896

Coutts A, Chen G, Stephens N, Hirst S, Douglas D, Eichholtz T, Khalil N (2001) Release of biologically active tgf-β from airway smooth muscle cells induces autocrine synthesis of collagen. American Journal of Physiology-Lung Cellular and Molecular Physiology 280(5):L999–L1008

Coxson H, Quiney B, Sin D, Xing L, McWilliams A, Mayo J, Lam S (2008) Airway wall thickness assessed using computed tomography and optical coherence tomography. American Journal of Respiratory and Critical Care Medicine 177(11):1201–1206

Dekkers BG, Pehlić A, Mariani R, Bos IST, Meurs H, Zaagsma J (2012) Glucocorticosteroids and β2-adrenoceptor agonists synergize to inhibit airway smooth muscle remodeling. Journal of Pharmacology and Experimental Therapeutics 342(3):780–787

Desmoulière A, Chaponnier C, Gabbiani G (2005) Tissue repair, contraction, and the myofibroblast. Wound repair and regeneration 13(1):7–12

Eskandari M, Pfaller MR, Kuhl E (2013) On the role of mechanics in chronic lung disease. Materials 6(12):5639–5658

Froese AR, Shimbori C, adn Mark Inman PSB, Obex S, Fatima S, Jenkins G, Gauldie J, Ask K, Kolb M (2016) Stretch-induced activation of transforming growth factor-β1 in pulmonary fibrosis. Am J Resp Crit Care Med 194:84–96

Gajarsa JJ, Kloner RA (2011) Left ventricular remodeling in the post-infarction heart: a review of cellular, molecular mechanisms, and therapeutic modalities. Heart Failure Reviews 16(1):13–21

Gasser T, Ogden R, Holzapfel G (2006) Hyperelastic modelling of arterial layers with distributed collagen fibre orientations. Journal of the Royal Society Interface 3:15–35

Gazzola M, Lortie K, Henry C, Mailhot-Larouche S, Chapman DG, Seow CY, Pare PD, King G, Boulet LP, Bosse Y (2017) Airway smooth muscle tone increases airway responsiveness in young healthy adults. American Journal of Respiratory and Critical Care Medicine 195:A4952

Ge Q, Poniris MH, Moir LM, Black JL, Burgess JK (2012) Combined beta-agonists and corticosteroids do not inhibit extracellular matrix protein production in vitro. Journal of allergy 2012:403,059

Gerarduzzi C, Di Battista JA (2017) Myofibroblast repair mechanisms post-inflammatory response: a fibrotic perspective. Inflammation Research 66(6):451–465

Gleason R, Humphrey J (2005) A 2d constrained mixture model for arterial adaptations to large changes in flow, pressure and axial stretch. Mathematical Medicine and Biology 22:347–369

Gleason R, Taber L, Humphrey J (2004) A 2-d model of flow-induced alterations in the geometry, structure, and properties of carotid arteries. American Society of Mechanical Engineers (ASME) Journal of Biomechanical Engineering 126:371–381

Grainge CL, Lau LC, Ward JA, Dulay V, Lahiff G, Wilson S, Holgate S, Davies DE, Howarth PH (2011) Effect of bronchoconstriction on airway remodeling in asthma. New England Journal of Medicine 364(21):2006–2015

Grinnell F, Ho CH (2002) Transforming growth factor β stimulates fibroblast–collagen matrix contraction by different mechanisms in mechanically loaded and unloaded matrices. Experimental cell research 273(2):248–255

Grytsan A, Eriksson TSE, Watton PN, Gasser TC (2017) Growth description for vessel wall adaptation: A thick-walled mixture model of abdominal aortic aneurysm evolution. Materials 10(9)

Guilbert TW, Morgan WJ, Zeiger RS, Mauger DT, Boehmer SJ, Szefler SJ, Bacharier LB, Lemanske Jr RF, Strunk RC, Allen DB, et al (2006) Long-term inhaled corticosteroids in preschool children at high risk for asthma. New England Journal of Medicine 354(19):1985–1997

Gunst S, Warner DO, Wilson T, Hyatt R (1988) Parenchymal interdependence and airway response to methacholine in excised dog lobes. Journal of Applied Physiology 65(6):2490–2497

Halwani R, Al-Muhsen S, Al-Jahdali H, Hamid Q (2011) Role of transforming growth factor–β in airway remodeling in asthma. American journal of respiratory cell and molecular biology 44(2):127–133

Harvey BC, Parameswaran H, Lutchen KR (2013) Can tidal breathing with deep inspirations of intact airways create sustained bronchoprotection or bronchodilation? Journal of applied physiology 115(4):436–445

Hassan M, Jo T, Risse PA, Tolloczko B, Lemiere C, Olivenstein R, Hamid Q, Martin JG (2010) Airway smooth muscle remodeling is a dynamic process in severe long-standing asthma. J Allergy Clin Immunol 125:1037–1045

Hill MR, Duan X, Gibson G, Watkins S, Robertson A (2012) A theoretical and non-destructive experimental approach for direct inclusion of measured collagen orientation and recruitment into mechanical models of the artery wall. Journal of Biomechanics 45:762–771

Hill MR, Simon MA, Valdez-Jasso D, Zhang W, Champion HC, Sacks MS (2014) Structural and mechanical adaptations of right ventricle free wall myocardium to pressure overload. Annals of Biomedical Engineering 42:2451–2465

Hiorns J, Jensen O, Brook B (2014) Nonlinear compliance modulates dynamic bronchoconstriction in a multiscale airway model. Biophysical Journal 107(12):3021–3033

Hiorns JE, Jensen OE, Brook BS (2016) Static and dynamic stress heterogeneity in a multiscale model of the asthmatic airway wall. Journal of Applied Physiology 121(1):233–247

Hirota JA, Nguyen TT, Schaafsma D, Sharma P, Tran T (2009) Airway smooth muscle in asthma: Phenotype plasticity and function. Pulmonary Pharmacology & Therapeutics 22(5):370 – 378

Hoffman BD, Grashoff C, Schwartz MA (2011) Dynamic molecular processes mediate cellular mechanotransduction. Nature 475(7356):316–323

Holgate ST (2011) The sentinel role of the airway epithelium in asthma pathogenesis. Immunological Reviews 242(1):205–219

Holzapfel G (2000) Nonlinear Solid Mechanics: A Continuum Approach for Engineering. Wiley, Chichester, New York

Holzapfel G, Ogden R (2010) Constitutive modelling of arteries. Proceedings of the Royal Society A: Mathematical, Physical and Engineering Sciences 466:1551–1597

Humphrey J, Rajagopal K (2002) A constrained mixture model for growth and remodeling of soft tissues. Mathematical Models and Methods in Applied Sciences (M3AS) 12:407–430

Humphrey J, Rajagopal K (2003) A constrained mixture model for arterial adaptations to a sustained step change in blood flow. Biomechanics and Modeling in Mechanobiology 2:109–126

Huyghe J, Janssen J (1997) Quadriphasic mechanics of swelling incompressible porous media. Int J Engng Sci 35:793–802

Ijpma G, Panariti A, Lauzon AM, Martin JG (2017) Directional preference of airway smooth muscle mass increase in human asthmatic airways. American Journal of Physiology-Lung Cellular and Molecular Physiology 312(6):L845–L854

James AL (2017) Airway remodeling in asthma: Is it fixed or variable? American Journal of Respiratory and Critical Care Medicine 195(8):968–970

James AL, Bai TR, Mauad T, Abramson MJ, Dolhnikoff M, McKay KO, Maxwell PS, Elliot JG, Green FH (2009) Airway smooth muscle thickness in asthma is related to severity but not duration of asthma. European Respiratory Journal 34(5):1040–1045

James AL, Elliot JG, Jones RL, Carroll ML, Mauad T, Bai TR, Abramson MJ, McKay KO, Green FH (2012) Airway smooth muscle hypertrophy and hyperplasia in asthma. American journal of respiratory and critical care medicine 185(10):1058–1064

Johnson JR, Wiley RE, Fattouh R, Swirski FK, Gajewska BU, Coyle AJ, Gutierrez-Ramos JC, Ellis R, Inman MD, Jordana M (2004) Continuous exposure to house dust mite elicits chronic airway inflammation and structural remodeling. American journal of respiratory and critical care medicine 169(3):378–385

Kariyawasam HH, Aizen M, Barkans J, Robinson DS, Kay AB (2007) Remodeling and airway hyperresponsiveness but not cellular inflammation persist after allergen challenge in asthma. American Journal of Respiratory and Critical Care Medicine 175(9):896–904

Kistemaker LEM, Bos ST, Mudde WM, Hylkema MN, Hiemstra PS, Wess J, Meurs H, Kerstjens HAM, Gosens R (2014) Muscarinic m3 receptors contribute to allergen-induced airway remodeling in mice. American Journal of Respiratory Cell and Molecular Biology 50(4):690–698

Kostenis E, Ulven T (2006) Emerging roles of dp and crth2 in allergic inflammation. Trends in Molecular Medicine 12(4):148–158

Kuo C, Lim S, King NJC, Johnston SL, Burgess JK, Black JL, Oliver BG (2011) Rhinovirus infection induces extracellular matrix protein deposition in asthmatic and nonasthmatic airway smooth muscle cells. American Journal of Physiology - Lung Cellular and Molecular Physiology 300(6):L951–L957

Lambert R, Wiggs B, Kuwano K, Hogg J, Pare P (1993) Functional significance of increased airway smooth muscle in asthma and copd. Journal of Applied Physiology 74(6):2771–2781

Lambert RK, Paré PD (1997) Lung parenchymal shear modulus, airway wall remodeling, and bronchial hyperresponsiveness. Journal of applied physiology 83(1):140–147

Lanir Y (1979) A structural theory for the homogeneous biaxial stress-strain relationships in flat collagenous tissues. Journal of Biomechanics 12:423–436

Lanir Y (1983) Constitutive equations for fibrous connective tissues. Journal of Biomechanics 16:1–12

LaPrad AS, Szabo T, Suki B, Lutchen K (2010) Tidal stretches do not modulate responsiveness of intact airways in vitro. Journal of Applied Physiology 109:295–304

Latourelle J, Fabry B, Fredberg JJ (2002) Dynamic equilibration of airway smooth muscle contraction during physiological loading. Journal of Applied Physiology 92(2):771–779

Lauzon AM, Bates JH (2000) Kinetics of respiratory system elastance after airway challenge in dogs. Journal of Applied Physiology 89(5):2023–2029

Leclere M, Lavoie-Lamoureux A, Joubert P, Relave F, Setlakwe EL, Beauchamp G, Couture C, Martin JG, Lavoie JP (2012) Corticosteroids and antigen avoidance decrease airway smooth muscle mass in an equine asthma model. American Journal of Respiratory Cell and Molecular Biology 47(5):589–596

Macklem PT (1996) A theoretical analysis of the effect of airway smooth muscle load on airway narrowing. American journal of respiratory and critical care medicine 153(1):83–89

Mailhot-Larouche S, Deschênes L, Gazzola M, Lortie K, Henry C, Brook BS, Morissette MC, Bossé Y (2018) Repeated airway constrictions in mice do not alter respiratory function. Journal of Applied Physiology DOI 10.1152/japplphysiol.01073.2017, URL https://doi.org/10.1152/japplphysiol.01073.2017, pMID: 29470147

Martinez-Lemus LA, Hill MA, Meininger GA (2009) The plastic nature of the vascular wall: A continuum of remodeling events contributing to control of arteriolar diameter and structure. Physiology 24(1):45–57

McKay KO, Wiggs BR, Paré PD, Kamm RD (2002) Zero-stress state of intra- and extraparenchymal airways from human, pig, rabbit, and sheep lung. Journal of Applied Physiology 92(3):1261–1266

McMillan S, Lloyd C (2004) Prolonged allergen challenge in mice leads to persistent airway remodelling. Clinical & Experimental Allergy 34(3):497–507

Miranda C, Busacker A, Balzar S, Trudeau J, Wenzel SE (2004) Distinguishing severe asthma phenotypes: role of age at onset and eosinophilic inflammation. Journal of Allergy and Clinical Immunology 113(1):101–108

Montesano R, Orci L (1988) Transforming growth factor beta stimulates collagen-matrix contraction by fibroblasts: implications for wound healing. Proceedings of the National Academy of Sciences 85(13):4894–4897

Moulton D, Goriely A (2011a) Circumferential buckling instability of a growing cylindrical tube. Journal of the Mechanics and Physics of Solids 59:525–537

Moulton D, Goriely A (2011b) Possible role of differential growth in airway wall remodeling in asthma. Journal of Applied Physiology 110:1003–1012

Naveed S, Clements D, Jackson DJ, Philp C, Billington CK, Soomro I, Reynolds C, Harrison TW, Johnston SL, Shaw DE, Johnson SR (2017) Matrix metalloproteinase-1 activation contributes to airway smooth muscle growth and asthma severity. American Journal of Respiratory and Critical Care Medicine 195(8):1000–1009

Noble PB, Jones RL, Cairncross A, Elliot JG, Mitchell HW, James AL, McFawn PK (2013) Airway narrowing and bronchodilation to deep inspiration in bronchial segments from subjects with and without reported asthma. Journal of Applied Physiology 114:1460–1471

Noble PB, Pascoe CD, Lan B, Ito S, Kistemaker LE, Tatler AL, Pera T, Brook BS, Gosens R, West AR (2014) Airway smooth muscle in asthma: Linking contraction and mechanotransduction to disease pathogenesis and remodelling. Pulmonary Pharmacology and Therapeutics 29:96–107

Oenema TA, Maarsingh H, Smit M, Groothuis GMM, Meurs H, Gosens R (2013) Bronchoconstriction induces tgf-β release and airway remodelling in guinea pig lung slices. PLOS ONE 8(6):1–9

Ojiaku MCA, Cao DG, Zhu DW, Yoo MEJ, Shumyatcher MM, Himes DBE, An DSS, Dr Reynold A Panettieri J (2017) Tgf-β1 evokes human airway smooth muscle cell shortening and hyperresponsiveness via smad3. American Journal of Respiratory Cell and Molecular Biology 0(ja):null, DOI 10.1165/rcmb.2017-0247OC, URL https://doi.org/10.1165/rcmb.2017-0247OC, pMID: 28984468, https://doi.org/10.1165/rcmb.2017-0247OC

Pelaia G, Renda T, Gallelli L, Vatrella A, Busceti MT, Agati S, Caputi M, mario Cazzola, Maselli R, Marsico SA (2008) Molecular mechanisms underlying airway smooth muscle contraction and proliferation: Implications for asthma. Computer Methods in Applied Mechanics and Engineering 314:222–268

Politi AZ, Donovan GM, Tawhai MH, Sanderson MJ, Lauzon AM, Bates JH, Sneyd J (2010) A multiscale, spatially distributed model of asthmatic airway hyper-responsiveness. Journal of theoretical biology 266(4):614–624

Pothen JJ, Poynter ME, Lundblad LKA, Bates JHT (2016) Dissecting the inflammatory twitch in allergically inflamed mice. AM J Physiol Lung Cell Mol Physiol 310:L1003–L1009

Ren JS (2013) Growth and residual stresses of arterial walls. Journal of Theoretical Biology 337(Supplement C):80 – 88, DOI https://doi.org/10.1016/j.jtbi.2013.08.008

Robertson A, Hill M, Li D (2011) Structurally motivated damage models for arterial walls- theory and application. In: Ambrosi D, Quarteroni A, Rozza G (eds) Modelling of Physiological Flows, Modeling, Simulation and Applications, vol 5, Springer-Verlag

Rodriguez EK, Hoger A, McCulloch AD (1994) Stress-dependent finite growth in soft elastic tissues. Journal of Biomechanics 27(4):455–467

Sacks M (2003) Incorporation of experimentally-derived fiber orientation into a structural constitutive model for planar collagenous tissues. American Society of Mechanical Engineers (ASME) Journal of Biomechanical Engineering 125:280–287

Saunders R, Siddiqui S, Kaur D, Doe C, Sutcliffe A, Hollins F, Bradding P, Wardlaw A, Brightling CE (2009) Fibrocyte localization to the airway smooth muscle is a feature of asthma. Journal of Allergy and Clinical Immunology 123(2):376 – 384

Silva PL, Passaro CP, Cagido VR, Bozza M, Dolhnikoff M, Negri EM, Morales MM, Capelozzi VL, Zin WA, Rocco PR (2008) Impact of lung remodelling on respiratory mechanics in a model of severe allergic inflammation. Respiratory Physiology & Neurobiology 160(3):239 – 248

Singh SR, Sutcliffe A, Kaur D, Gupta S, Desai D, Saunders R, Brightling CE (2014) Ccl2 release by airway smooth muscle is increased in asthma and promotes fibrocyte migration. Allergy 69(9):1189–1197

Sjöberg L, Nilsson AZ, Lei Y, Gregory J, Adner M, Nilsson G (2017) Interleukin 33 exacerbates antigen driven airway hyperresponsiveness, inflammation and remodeling in a mouse model of asthma. Scientific Reports 7

Skalak R (1980) Growth as a finite displacement field. Proceedings of the IUTAM Symposium on Finite Elasticity pp 347–355

Smith PG, Janiga KE, Bruce MC (1994) Strain increases airway smooth muscle cell proliferation. American Journal of Respiratory Cell and Molecular Biology 10(1):85–90

Smith PG, Tokui T, Ikebe M (1995) Mechanical strain increases contractile enzyme activity in cultured airway smooth muscle cells. American Journal of Physiology - Lung Cellular and Molecular Physiology 268(6):L999–L1005

Smith PG, Moreno R, Ikebe M (1997) Strain increases airway smooth muscle contractile and cytoskeletal proteins in vitro. American Journal of Physiology - Lung Cellular and Molecular Physiology 272(1):L20–L27

Strunk RC (2007) Childhood asthma management program: Lessons learned. The Journal of Allergy and Clinical Immunology 119(1):36–42

Swartz M, Tschumperlin D, Kamm R, Drazen J (2001) Mechanical stress is communicated between different cell types to elicit matrix remodeling. PNAS 98:6180–6185

Tatler AL, Jenkins G (2012) Tgf-β activation and lung fibrosis. Proceedings of the American Thoracic Society 9(3):130–136

Tatler AL, John AE, Jolly L, Habgood A, Porte J, Brightling C, Knox AJ, Pang L, Sheppard D, Huang X, Jenkins G (2011) Integrin $\alpha v\beta 5$-mediated tgf-β activation by airway smooth muscle cells in asthma. The Journal of Immunology 187(11):6094–6107

Truesdell C, Noll W (1965) The Non-Linear Field Theories of Mechanics. Springer-Verlag, Berlin, Germany

Truesdell C, Toupin R (1960) The Classical Field Theories, Springer, Heidelberg. Handbuch der Physik

Tschumperlin DJ, Drazen J (2001) Mechanical stimuli to airway remodeling. American Journal of Respiratory and Critical Care Medicine 164:S90–S94

Tschumperlin DJ, Drazen J (2006) Chronic effects of mechanical force on airways. Annual Review of Physiology 68:563–583

Tschumperlin DJ, Dai G, Maly IV, Kikuchi T, Laiho LH, McVittie AK, Haley KJ, Lilly CM, So PT, Lauffenburger DA, et al (2004) Mechanotransduction through growth-factor shedding into the extracellular space. Nature 429(6987):83–86

Valentin A, Humphrey J, Holzapfel G (2013) A finite element-based constrained mixture implementation for arterial growth, remodeling, and adaptation: Theory and numerical verification. International Journal for Numerical Methods in Biomedical Engineering 29:822–849

Wang I, Politi AZ, Tania N, Bai Y, Sanderson MJ, Sneyd J (2008) A mathematical model of airway and pulmonary arteriole smooth muscle. Biophysical journal 94(6):2053–2064

Wenzel SE (2012) Asthma phenotypes: the evolution from clinical to molecular approaches. Nature medicine 18(5):716–725

Williamson JP, McLaughlin RA, Noffsinger WJ, James AL, Baker VA, Curatolo A, Armstrong JJ, Regli A, Shepherd KL, Marks GB, Sampson DD, Hillman DR, Eastwood PR (2011) Elastic properties of the central airways in obstructive lung diseases measured using anatomical optical coherence tomography. American Journal of Respiratory and Critical Care Medicine 183(5):612–619

Wipff PJ, Rifkin DB, Meister JJ, Hinz B (2007) Myofibroblast contraction activates latent tgf-$\beta 1$ from the extracellular matrix. J Cell Bio 179:1311–1323

Woolley KL, Gibson PG, Carty K, Wilson AJ, Twaddell SH, Woolley MJ (1996) Eosinophil apoptosis and the resolution of airway inflammation in asthma. American journal of respiratory and critical care medicine 154(1):237–243

Wright DB, Trian T, Siddiqui S, Pascoe CD, Johnson JR, Dekkers BG, Dakshinamurti S, Bagchi R, Burgess JK, Kanabar V, Ojo OO (2013) Phenotype modulation of airway smooth muscle in asthma. Pulmonary Pharmacology & Therapeutics 26(1):42–49

Zhu Z, Homer RJ, Wang Z, Chen Q, Geba GP, Wang J, Zhang Y, Elias JA (1999) Pulmonary expression of interleukin-13 causes inflammation, mucus hypersecretion, subepithelial fibrosis, physiologic abnormalities, and eotaxin production. Journal of Clinical Investigation 103(6):779
